# Plastic-inhabiting fungi in marine environments and PCL degradation activity

**DOI:** 10.1007/s10482-022-01782-0

**Published:** 2022-10-14

**Authors:** Sung Hyun Kim, Jun Won Lee, Ji Seon Kim, Wonjun Lee, Myung Soo Park, Young Woon Lim

**Affiliations:** 1 School of Biological Sciences and Institution of Microbiology, Seoul National University, Seoul, 08826 Republic of Korea; 2 Department of Crops and Forestry, Korea National College of Agriculture and Fisheries, Jeonju, 54874 Republic of Korea

**Keywords:** Fungal diversity, Marine fungi, Phylogenetic analysis, Plastic degradation, Polycaprolactone

## Abstract

Plastic waste has a negative impact on marine ecosystems and the quantity of this source of anthropogenic pollution continues to increase. Several studies have investigated plastic biodegradation using various microorganisms. In this study, we isolated fungi from polyethylene terephthalate (PET) waste on Korean seacoasts and evaluated their ability to degrade plastic by comparing the diameters of the clear zones they formed on polycaprolactone (PCL) agar. We isolated 262 strains from 47 plastic waste sources and identified 108 fungal species via molecular methods. The PCL agar assay revealed that 87 species presented with varying degrees of PCL degradation capacity. Among them, certain fungal species were strong PCL degraders. The present study demonstrated the possibility that some fungi inhabiting plastic could potentially degrade it in the marine environment. We believe that the discoveries made herein lay theoretical and practical foundations for the development of novel bioremediation systems for marine plastispheres and help mitigate the environmental pollution issues related to plastic wastes.

**Supplementary Information:**

The online version contains supplementary material available at 10.1007/s10482-022-01782-0.

## Introduction

Plastic products are extensively and widely used, and the plastic polymer compositions vary depending on the intended applications of the products they are used to fabricate. Common plastics include high-density polyethylene (HDPE), low-density polyethylene (LDPE), and polyethylene terephthalate (PET) (Plastics Europe, 2021). As of 2020, approximately 367 million tonnes of plastic products were produced. The plastic production volume has substantially increased since the 1990s (Plastics Europe, 2021). However, only about 9% of all plastic waste is recycled, and as much as 60% of it is buried in a landfill or discarded as litter in terrestrial environments (Geyer et al. 2017). Most marine plastic waste consists of improperly disposed terrestrial plastic waste that has entered the oceans via rivers, wastewater outflows, wind, and tides. In 2010, 4–12 million tonnes of marine plastic waste originated from land (Jambeck et al. 2015). Additionally, about 1.15–2.41 million tonnes of marine plastic waste originated from river (Lebreton et al. 2017).

Plastic waste accumulation has had a direct and indirect negative impact on marine ecosystems. Plastic pieces are ingested by marine animals and damage their internal organs (Ahrendt et al. 2020; Wright et al. 2013). Furthermore, wave action, weathering, and other processes break down plastic pieces into microplastics which are the plastic wastes that were degraded into smaller fragments and fibers (Thompson et al. 2004), causing other more hazards. Microplastics float on ocean surfaces and contain Persistent Organic Pollutants (POPs), which are endocrine disruptors that hinder the survival rate of marine organisms (Rios et al. 2007). Microplastics also transport microbial pathogens and alien species, hazardous to marine ecosystems in other regions (Arias-Andres et al. 2018; Beloe et al. 2022; Bowley et al. 2021). Thus, plastic in marine ecosystems has become a serious environmental issue. However, there are no policies or solutions in place that effectively mitigate the plastic waste problem. Recent studies have explored chemical degradation, recycling, and biodegradation as potential marine plastic waste remediation measures.

Much research attention has been directed toward plastic waste degradation by microorganisms. Plastic waste has persisted in natural environments for decades. Plastic debris that is inhabited and partially decomposed by the microbial community is now referred to as the “plastisphere” (Zettler et al. 2013) and numerous different microorganisms are found in it (Hirota et al. 2021; Amaral-Zettler et al. 2020). Some of them were reported to degrade plastic by various kinds of tests (Badahit et al. 2018; Sangeetha Devi et al. 2019; Hou et al. 2022; Kumari et al. 2019; Muhonja et al. 2018; Yamada-Onodera et al. 2001): and enzymatic activities involved in plastic degradation have been investigated extensively (Temporiti et al. 2022). Nevertheless, prior research has focused mainly on plastic-decomposing bacteria. Fungi comprise only about 3% of all eukaryotic organisms in the plastisphere, although they play a vital role as decomposers in the environment (Rogers et al., 2020). Numerous plastic-degrading fungi have been detected and identified in the landfill (terrestrial) plastisphere including *Aspergillus* spp. (Cosgrove et al. 2007; Muhonja et al. 2018; Zahra et al. 2010), *Fusarium* spp. (Kanelli et al. 2015; Zahra et al. 2010), and *Penicillium simplissimum* (Yamada-Onodera et al. 2001). Previous studies on plastic-degrading fungi in marine environments concentrated primarily on several specific taxa such as *Aspergillus* sp. (Sarkhel et al. 2020) and *Zalerion maritimum* (Paço et al. 2017).

Previously, we isolated different fungi from various substrates in marine environments such as sailfin eggs (Park et al. 2018) and microalgae (Lee et al. 2019; Park et al. 2016). Many of these fungi had high enzymatic activity (Lee et al., 2019; Park et al. 2015a, 2019). Since these studies detected the fungal ability to degrade complex organic matter, it was expected that plastic-isolated fungi could decompose plastic substrates. A metabarcoding analysis revealed that a wide array of fungi survived on plastics collected from seawater (Lacerda et al. 2020, Davidov et al. 2020) and the sea floor (De Tender et al. 2017). We hypothesized that different fungi can inhabit plastic waste and most of them actively participate in plastic degradation. In the present study, therefore, we investigated fungal diversity in PET waste collected from seacoasts and used a polycaprolactone (PCL) degradation assay to evaluate their capacity to degrade plastic. PCL is a biopolymer that has been extensively used in biodegradation research as a surrogate for non-degradable polymers. Its usage in fungal incubation varies from film/sheet form (Benedict et al. 1983; Fukushima et al. 2010), or agar from as emulsified substance (Lee et al. 2021).

## Materials and methods

### Sampling

**Fig. 1:**
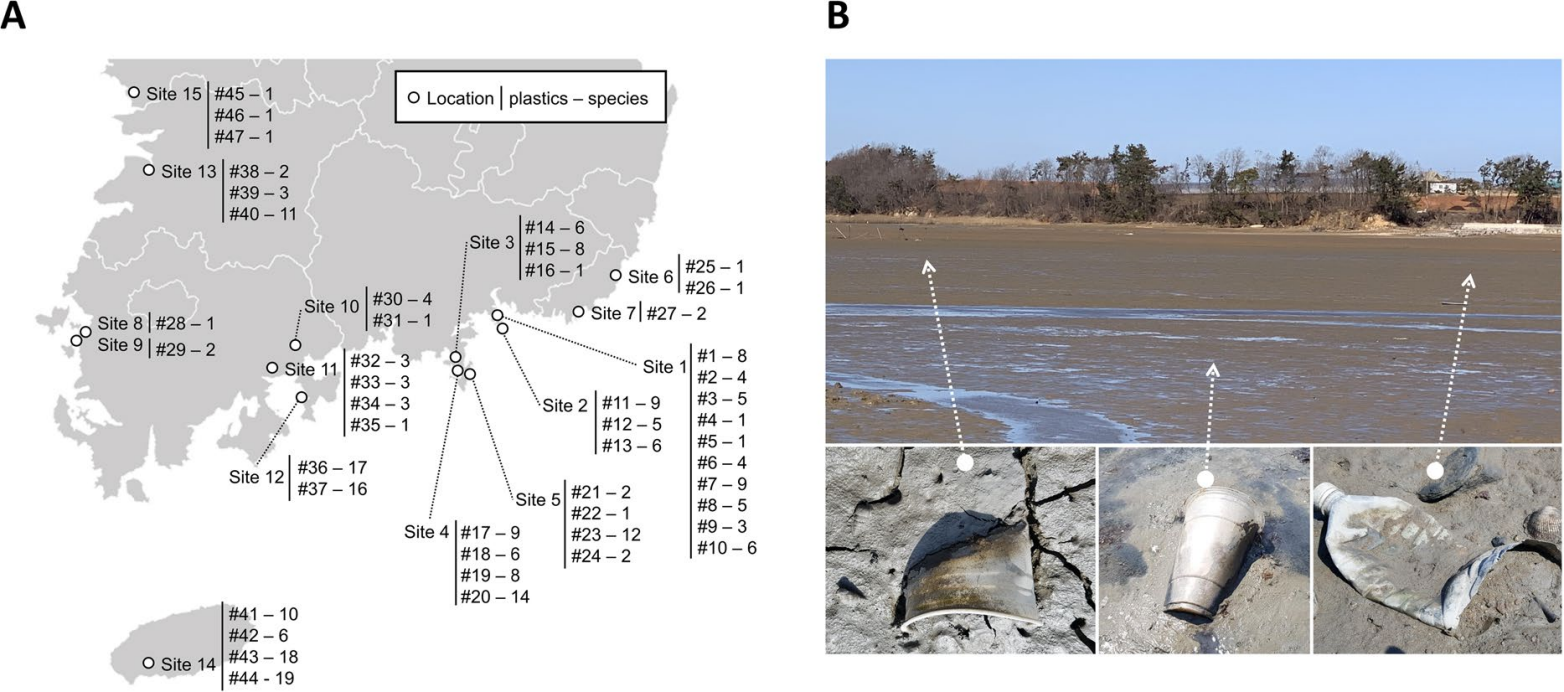
Sampling site of plastic waste along Korean seacoasts and number of fungal strain isolated (A). Example of plastic wastes collected from mudflats and sand (B)

Forty-seven PET wastes such as PET bottles and PET cups were collected from 15 sites along the western and southern sea coast of the Republic of Korea in April, 2018 (Fig. 1). We collected PET bottles and PET cups with intact shape to prevent wrong sample collection. The PET surfaces were cleansed of debris by washing with artificial seawater (ASW). Each PET waste sample was cut with sterilized scissors into 27 pieces each 1 cm^2^ in area. To isolate the fungi, nine pieces per sample were placed in dichloran rose bengal chloramphenicol agar (DRBC; Difco, Sparks, MD, USA), glycerol yeast extract agar (GYA; 1 g L^−1^ glucose, 0.1 g L^−1^ yeast extract, 0.5 g L^−1^ peptone, and 15 g L^−1^ agar), and potato dextrose agar (PDA; Difco, Sparks, MD, USA) supplemented with ASW (purified from seawater in South Korea; salinity = 32.0%). The plates were incubated at 25 °C for 7–14 d. Pure fungal colonies were then transferred to new PDA + ASW plates. Pure fungal strains were stored in 20% (v/v) glycerol at –80 °C and deposited in the Seoul National University Fungus Collection (SFC).

### Molecular identification

The fungal isolates on PDA were grouped according to their morphological characteristics such as texture, color, colony size, and sporulation. At least one strain was selected from each group for molecular identification. Genomic DNA was extracted by a modified cetyltrimethylammonium bromide (CTAB) method (Rogers and Bendich 1994). PCR amplification of the internal transcribed spacer (ITS) region was performed using ITS1F/ITS4 primers (Gardes and Bruns 1993; White et al. 1990) and AccuPower PCR Master Premix (Bioneer Co., Daejeon, Republic of Korea). All representative strains were identified down to the genus level based on their ITS sequences. The strains within certain genera were identified to the species level using various protein-coding gene analyses and different primer sets. Actin (*act*) was amplified using ACT-512F/ACT-783R (Carbone and Kohn 1999) or ACT1Fd/ACT1Rd (Aveskamp et al. 2009; Groenewald et al. 2013) whilst β-tubulin (*BenA*) was amplified using Bt2a/Bt2b (Glass and Donaldson 1995). PCR was performed in a C1000 Thermal Cycler (Bio-Rad Laboratories, Hercules, CA, USA) under previously described conditions (Park et al. 2015b). PCR amplicons were checked with 1% agarose gel and purified with an Expin™ PCR Purification Kit (GeneAll Biotechnology, Seoul, Korea) according to the manufacturer's instructions. DNA was sequenced at Macrogen (Seoul, Republic of Korea) in an ABI PRISM 3700 Genetic Analyzer (Life Technologies, Gaithersburg, MD, USA).

All sequences were proofread and edited with MEGA7 (Kumar et al. 2016) and deposited in GenBank (Supplementary Table 2). For the phylogenetic analysis, the type sequences of the reference species were retrieved from GenBank (Supplementary Table 3) and aligned with the sample sequences for each locus with MAFFT v. 7 (Katoh and Standley 2013) using the default parameters. Maximum likelihood phylogenetic analyses were performed on each gene (ITS, *act*, *BenA*) using RAxML (Stamatakis 2006), the GTRGAMMA evolution model, and 1,000 bootstrap replicates.

### PCL degradation test on agar plate

The PET-degrading ability was determined for one to nine representative strains of each fungal species (Table 1) by measuring the clear zones formed in polycaprolactone (PCL) media. The PCL agar was prepared according to a previously reported method (Lee et al. 2021) and consisted of a 1% (w/v) emulsified PCL suspension (pellet form, 3 mm in diameter; Sigma-Aldrich., St. Louis, MO, USA) in acetone plus distilled water (10% of acetone volume). The PCL suspension was added to an autoclaved medium comprising a 0.8% (w/v) yeast nitrogen base (Difco-Becton Dickinson, Broken Bow, NE, USA), 1.5% (w/v) agar, and distilled water, then poured into 90 mm-Petri dishes.

**Table 1:** Fungi isolated from PET wastes and their PCL degradation assay results. Fungal orders are in bold font. Averages and SD of clear zone lengths are presented and categorized into four levels

Species	Total no. strains	PCL tested strains	Clear zone length (mm)	Degradation level*
Amphisphaeriales				
* Morinia* cf. *acaciae*	1	1	1.55	( +)
* Neopestalotiopsis* sp.	2	1	0	0
* Pestalotiopsis* cf. *anacardiacearum*	1	1	0.88	( +)
* Pestalotiopsis* cf. *australasiae*	2	1	0.34	( +)
* Pestalotiopsis* sp.	2	1	0.9	( +)
* Pestalotiopsis thailandica*	1	1	1.35	( +)
Botryosphaeriales				
* Botryosphaeria dothidea*	1	1	0.37	( +)
* Sphaeropsis sapinea*	1	1	6.94	(+ +)
Capnodiales				
* Neodevriesia* cf. *metrosideri*	1	1	6.08	(+ +)
Cladosporiales				
* Cladosporium allicinum*	2	1	13.92	(+ + +)
* Cl. anthropophilum*	7	1	9.74	(+ +)
* Cl.* cf. *halotolerans*	2	1	8.6	(+ +)
* Cl.* cf. *cladosporioides*	1	1	8.22	(+ +)
* Cl. funiculosum*	1	1	1.66	( +)
* Cl. halotolerans*	1	1	2.46	( +)
* Cl. perangustum*	2	2	6.76 (± 4.19)	(+ +)
* Cl. pseudocladosporioides*	3	2	7.74 (± 6.84)	(+ +)
* Cl. ramotenellum*	16	6	4.85 (± 2.17)	( +)
* Cl. rectoides*	5	2	10.34 (± 3.28)	(+ + +)
* Cl. tenuissimum*	7	3	10.21 (± 0.82)	(+ + +)
* Cl. xanthochromaticum*	2	1	11.37	(+ + +)
* Cl. xylophilum*	4	2	0.57 (± 0.11)	( +)
Diaporthales				
* Diaporthe* cf. *arecae*	3	2	1.33 (± 0.4)	( +)
* Diaporthe* cf. *hungariae*	5	2	2.48 (± 0.95)	( +)
* Diaporthe* cf. *pseudooculi*	1	1	2.18	( +)
*Diaporthe* cf. *sojae*	1	1	1.09	( +)
* Cytospora ceratosperma*	1	1	6.17	(+ +)
Dothideales				
* Aureobasidium melanogenum*	1	1	8.28	(+ +)
* Au. namibiae*	1	1	6.24	(+ +)
* Au. pullulans*	2	1	0.56	( +)
Eurotiales				
* Aspergillus ochraceus*	1	1	2.19	( +)
* As. oryzae*	1	1	4.8	( +)
* As. tritici*	1	1	2.03	( +)
* Penicillium charlesii*	2	1	0	0
* Pe. commune*	4	2	0 (± 0)	0
* Pe. crustosum*	3	1	0	0
* Pe. echinulatum*	1	1	0.37	( +)
* Pe. expansum*	1	1	0	0
* Pe. exsudans*	1	1	0	0
* Pe. javanicum*	1	1	0	0
* Pe. oxalicum*	1	1	0	0
* Pe. roqueforti*	1	1	0.07	( +)
* Talaromyces rugulosus*	1	1	3.31	( +)
Glomerellales				
* Plectosphaerella cucumerina*	1	1	0.39	( +)
Helotiales				
* Botrytis cinerea*	1	1	1.03	( +)
Hypocreales				
* Acremonium* cf. *fuci*	12	3	0 (± 0)	0
* Ac. fuci*	8	2	0 (± 0)	0
* Fusarium equiseti*	4	2	0.63 (± 0.21)	( +)
* Fusarium fujikuroi*	1	1	0.73	( +)
Hypocreales sp.	1	1	2.43	( +)
* Parasarocladium* cf. *gamsii*	2	2	3.38 (± 4.08)	( +)
* Parengyodontium album*	1	1	0	0
* Sarocladium strictum*	1	1	7.63	(+ +)
* Trichoderma harzianum*	1	1	0.41	( +)
* Tr. fomiticola*	1	1	0.3	( +)
Mycosphaerellales				
* Phaeophleospora eucalypticola*	2	1	13.96	(+ + +)
Pleosporales				
* Alternaria alternata*	21	8	6.14 (± 3.28)	(+ +)
* Al.* cf. *rosae*	2	1	5.82	(+ +)
* Al. chlamydospora*	3	1	1.7	( +)
* Didymella* cf. *macrophylla*	1	1	1.28	( +)
Didymosphaeriaceae sp. 1	1	1	6.1	(+ +)
Didymosphaeriaceae sp. 2	1	1	3.33	( +)
* Epicoccum* cf. *duchesneae*	3	1	3.58	( +)
* Epicoccum* cf. *hordei*	1	1	0	0
* Epicoccum* cf. *sorghinum*	1	1	2.65	( +)
* Epicoccum dendrobii*	2	1	6.26	(+ +)
* Epicoccum duchesneae*	3	2	0.43 (± 0.61)	( +)
* Epicoccum sorghinum*	2	1	1.5	( +)
* Epicoccum* sp.	1	1	0.35	( +)
* Epicoccum tritici*	2	1	2.34	( +)
* Juxtiphoma* cf. *eupyrena*	1	1	0	0
* Kalmusia araucariae*	1	1	0	0
* Neocamarosporium betae*	3	1	0	0
* Neocamarosporium solicola*	2	1	0.55	( +)
* Neocamarosporium* sp.	3	2	0.86 (± 0.72)	( +)
* Neodidymelliopsis* cf. *longicolla*	6	3	2.61 (± 3.37)	( +)
* Neodidymelliopsis longicolla*	2	1	0.77	( +)
* Neosetophoma* cf. *poaceicola*	1	1	2.31	( +)
* Neosetophoma poaceicola*	1	1	0.94	( +)
* Neosetophoma rosigena*	1	1	0.58	( +)
* Nothophoma quercina*	1	1	7.67	(+ +)
* Paraconiothyrium brasiliense*	1	1	0	0
* Paradendryphiella arenariae*	14	3	0.17 (± 0.3)	( +)
* Paraphoma radicina*	1	1	0.77	( +)
* Parathyridaria* cf. *tyrrhenica*	2	2	0.73 (± 1.03)	( +)
* Phaeosphaeria culmorum*	1	1	0	0
* Phaeosphaeria spartinicola*	1	1	5.27	(+ +)
* Phaeosphaeria oryzae*	1	1	6.42	(+ +)
Pleosporaceae sp. 1	1	1	0.42	( +)
Pleosporaceae sp. 2	1	1	2.07	( +)
Pleosporales sp. 1	12	2	0 (± 0)	0
Pleosporales sp. 2	3	2	1.33 (± 1.15)	( +)
Pleosporales sp. 3	1	1	0.93	( +)
Pleosporales sp. 4	2	2	3.37 (± 1.52)	( +)
* Pyrenochaetopsis microspora*	2	1	1.24	( +)
* Pyrenochaetopsis paucisetosa*	1	1	0.69	( +)
* Remotididymella* cf. *capsici*	3	2	0.51 (± 0.72)	( +)
* Remotididymella* sp.	1	1	2.78	( +)
* Stemphylium lycopersici*	1	1	1	( +)
* St. vesicarium*	2	2	1.66 (± 0.07)	( +)
Sordariales				
* Chaetomium globosum*	1	1	0.93	( +)
Thelebolales				
* Pseudogymnoascus pannorum*	2	1	0.89	( +)
Xylariales				
* Eutypella* cf. *persica*	1	1	0.69	( +)
Others (incertae sedia)				
* Apiospora marii*	1	1	0.51	( +)
* Ap. rasikravindrae*	1	1	0.85	( +)
* Nigrospora* cf. *oryzae*	2	1	0	0
* Sedecimiella taiwanensis*	1	1	0.19	( +)
* Septoriella* cf. *hubertusii*	1	1	0.33	( +)

^*^0 mm: (0), 0 < ( +) ≤ 5 mm, 5 < (+ +) ≤ 10 mm, 10 < (+ + +) ≤ 15 mm

Representative strains of each fungal species identified were inoculated with a 4-mm hole punch at the center of each agar plate. Clear zone formation was evaluated by measuring the distance between the margin of the clear (transparent) zone and that of the colony after 7 d incubation at 25 °C. All clear zones were measured in triplicate and averaged. PCL degradation by each species was determined from the averages of the clear zone lengths of all representative strains of the same species. For species with multiple tested strains, the standard deviations of the average clear zone lengths of all strains within the same species were also calculated.

## Results

### Identification and diversity analysis

**Fig. 2:**
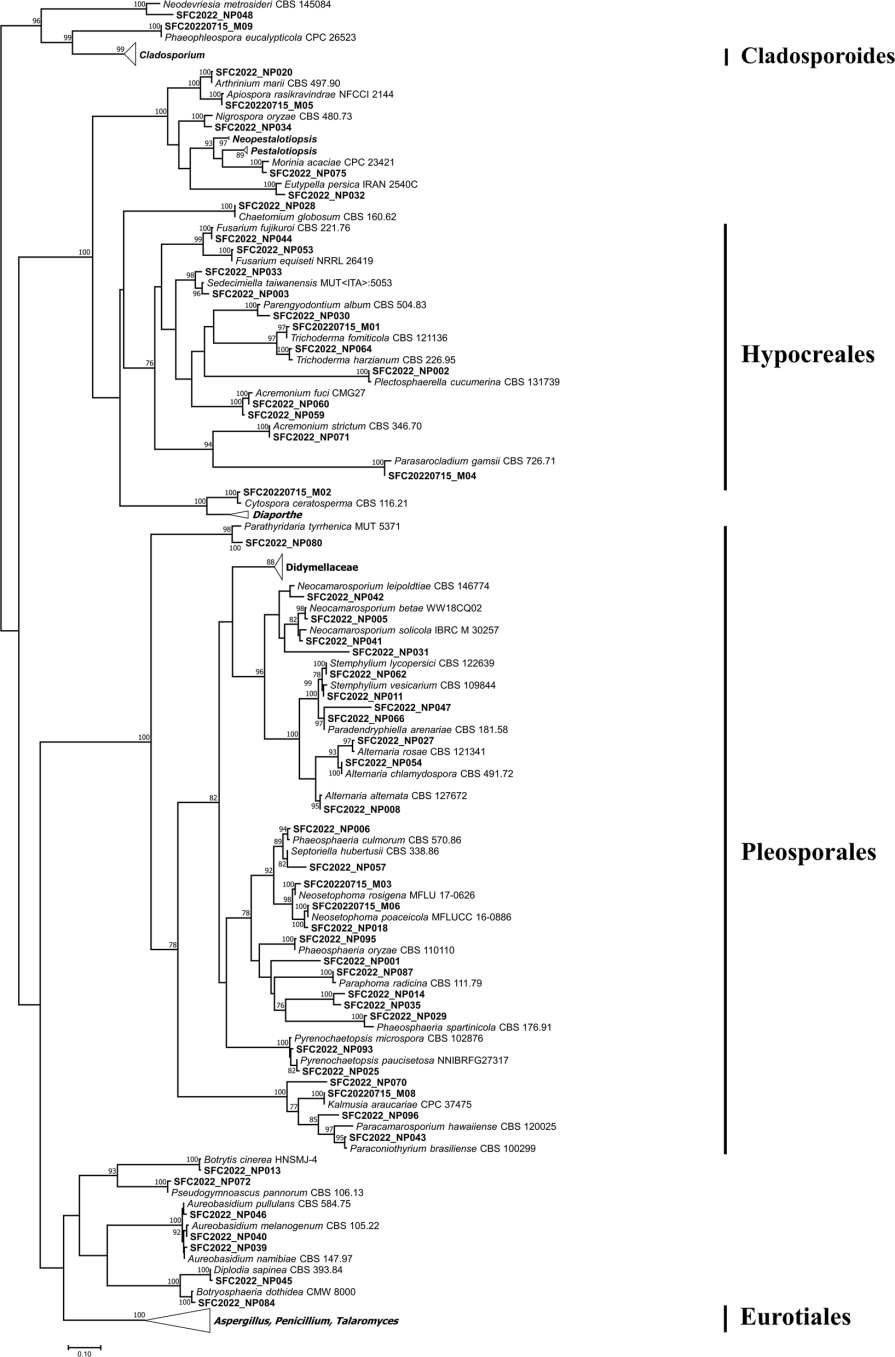
Phylogenetic tree of fungi isolated from marine plastic waste based on ML analysis of ITS. Bootstrap scores > 70 are presented at nodes. Scale bar indicates the number of nucleotide substitutions per site. Representative strains of each taxon based on ITS sequences are shown in bold font

A total of 262 fungal strains were isolated from 47 PET wastes. Multiple strains of the same species derived from a single PET waste were treated as a single strain. One to nineteen fungal strains were isolated per PET. Depending on the isolation medium used (DRBC, GYA, or PDA), different numbers of fungal strains were isolated from the same PET waste (Fig. 1, Supplementary Table S1). All fungal strains were grouped into 108 taxa based on their morphological features and ITS sequencing results (Fig. 2). Forty-seven taxa were identified to the species level based on their protein-coding genes. The actin gene was used to identify *Cladosporium* species whilst the β-tubulin gene was used to identify *Aspergillus*, *Diaporthe*, *Didymella*, *Epicoccum*, *Juxtiphoma*, *Neodidymelliopsis*, *Nothophoma*, *Penicillium*, *Pestalotiopsis*, *Remotididymella*, and *Talaromyces* species (Table 1, Supplementary Figure S1). Based on the ITS sequences alone, 47 taxa were confirmed to the species level whilst 14 others were identified to the genus, family, and order levels.

**Fig. 3:**
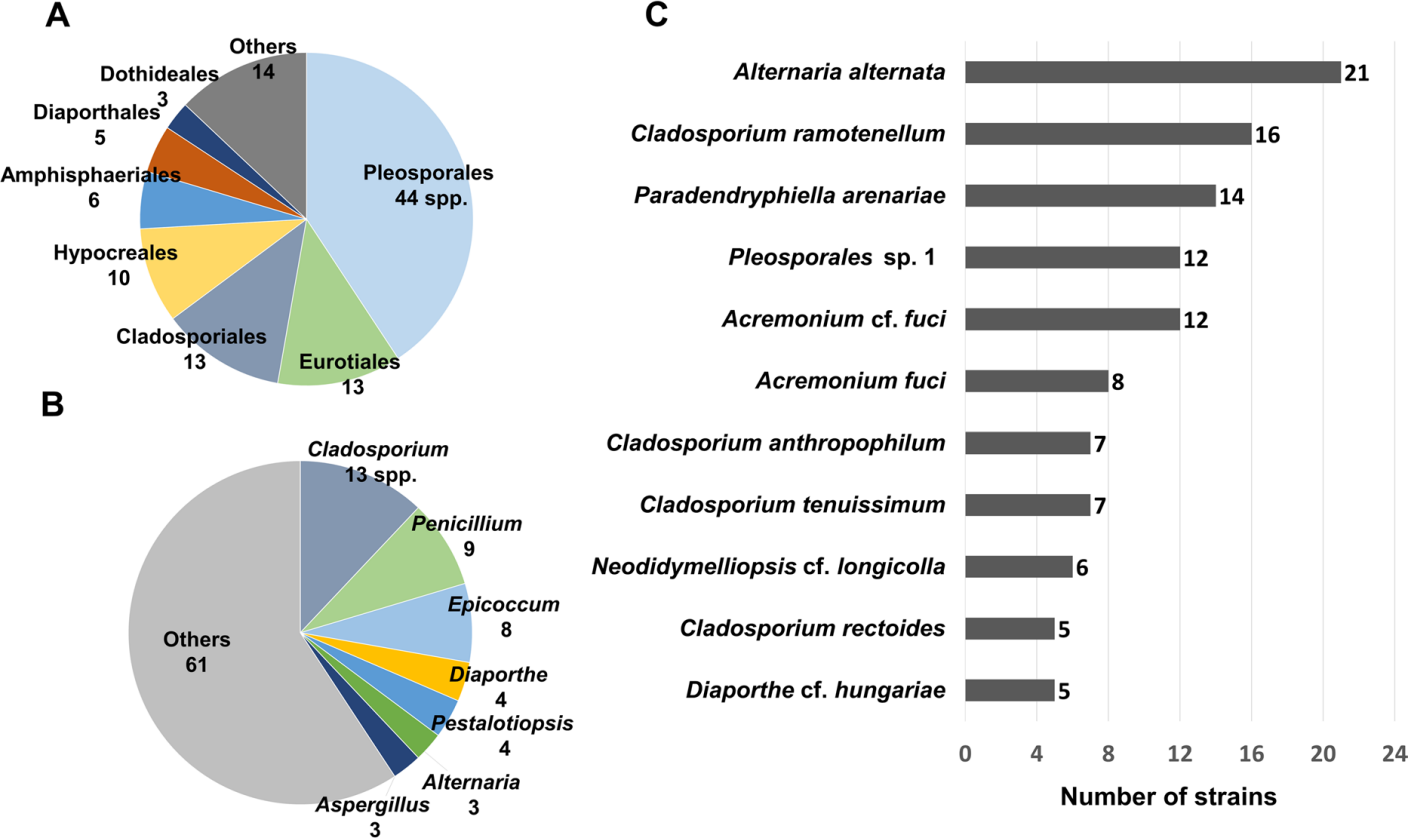
Proportions of fungi isolated from plastic waste. Order (A), genus (B), and species (C) levels

All 108 species detected belonged to the Ascomycota and were classified into 15 orders and 46 genera (Table 1, Fig. 2). Pleosporales was the dominant order and included 44 species. It was followed by Cladosporiales and Eurotiales with 13 species each (Fig. 3A). Cladosporiales only included the genus *Cladosporium* whereas Eurotiales comprised the genera *Penicillium*, *Aspergillus*, and *Talaromyces*. The latter two included three and one species, respectively, and nine *Penicillium* species were identified (Fig. 3B, Table 1). Eleven different species were isolated from at least five PET wastes (Fig. 3C). *Alternaria alternata* was isolated from 21 different PET waste sources followed by *Cladosporium ramotenellum* (16 PET wastes) and *Paradendryphiella arenariae* (14 PET wastes).

### Fungal PCL degradation activity

**Fig. 4:**
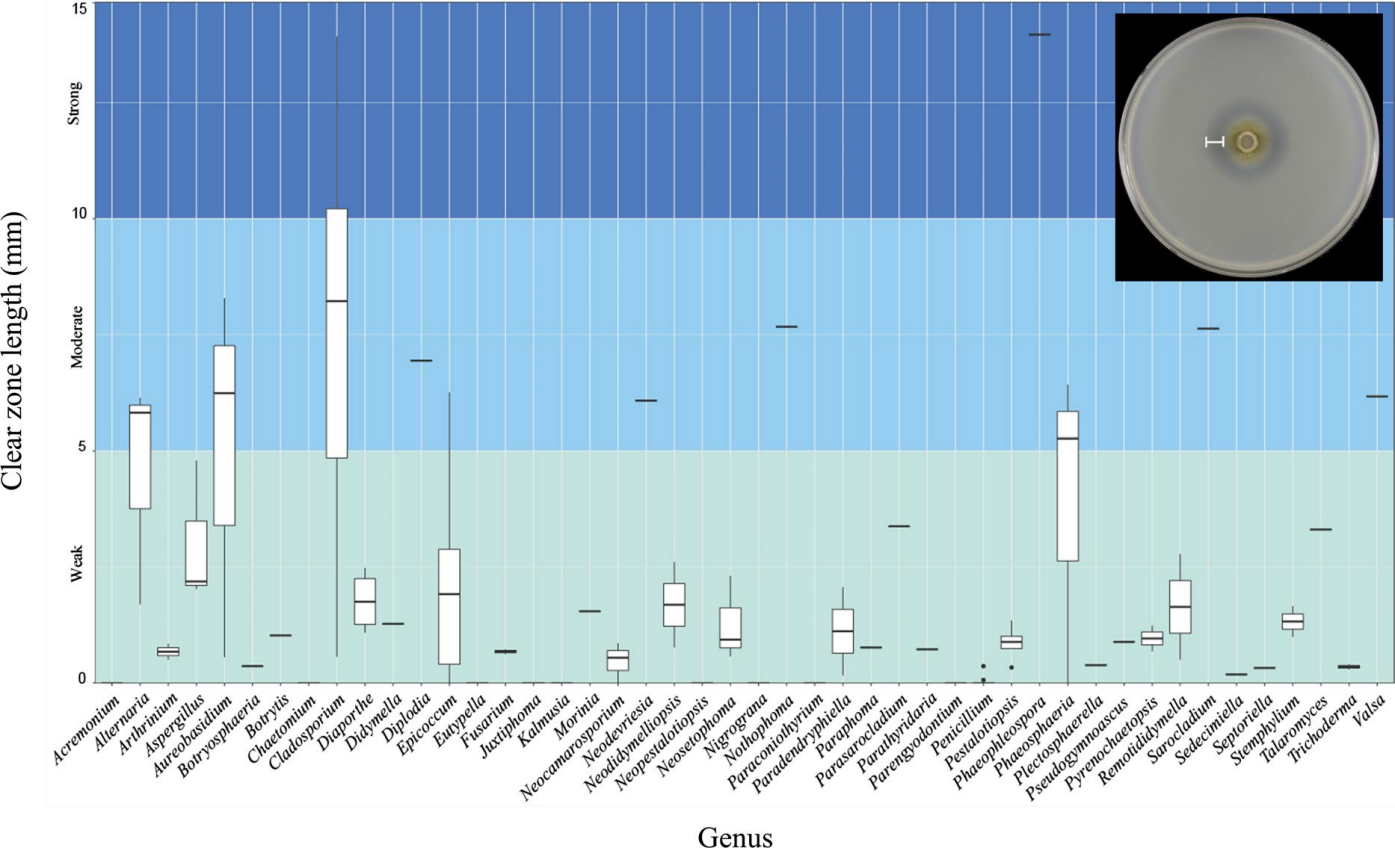
Boxplot of PCL-degrading ability of all tested strains with the genera detected in the present study. Boxplot constructed based on average clear zone length of each species. Clear zone lengths are distances between colony margins and transparent areas. Inset: photograph of *Cladosporium rectoides* (SFC2022_NP016) culture

A PCL degradation test was performed on 146 representative strains of 108 species (Supplementary Table S3). The clear zone lengths of the fungal strains were in the range of 0–13.96 mm. Based on the average clear zone lengths, fungal PCL degradation ability was categorized into four levels, namely, no degradation (0 mm: 0), weak (0 < ( +) ≤ 5 mm), moderate (5 < (+ +) ≤ 10 mm), and strong (10 < (+ + +) ≤ 15 mm) (Fig. 4). Five species exhibited strong PCL degradation, 18 species showed moderate PCL degradation, 64 species presented with weak PCL degradation, and 21 species did not degrade PCL at all (Table 1). There was also intraspecies variation. The PCL degradation capacities of eight *Alternaria alternata* strains ranged from 12.88 mm (NP321) to 3.32 mm (NP044). In most cases, however, all tested strains of the same species were similar in terms of their PCL degradation ability.

*Phaeophleospora eucalypticola* had the strongest PCL degradation ability (clear zone length = 13.96 mm). Four *Cladosporium* species also showed strong PCL degradation activity. *Cladosporium allicinum* had the widest clear zone (13.92 mm) followed by *C. xanthochromaticum* (11.37 mm), *C. rectoides* (10.34 mm), and *C. tenuissimum* (10.21 mm). Seventeen species of moderate PCL-degrading fungi were classified into ten genera including two *Alternar*i*a* spp., two *Aureobasidium* spp., two *Phaeosphaeria* spp., five *Cladosporium* spp., and each one species of *Cytospora*, *Epicoccum*, *Neodevriesia*, *Nothophoma, Sarocladium, Sphaeropsis* (Table 1). *Cladosporium* species showed relatively high PCL degradation activity among the moderate PCL degraders.

Weak PCL-degrading fungi included 64 species. They were classified as 30 genera (Table 1). *Didymella*, *Epicoccum*, and *Remotiodidymella* (Didymellaceae) showed relatively weak PCL degradation as no strain produced a clear zone wider than 5 mm. The species in the Eurotiales exhibited very weak PCL degradation ability. None of the *Penicillium* strains produced clear zones wider than 1 mm (Fig. 4, Table 1). *Aspergillus* and *Talaromyces* showed higher PCL-degrading activity than *Penicillium*. The lengths of the average clear zones produced by *Aspergillus* and *Talaromyces* were 2.30 and 3.31 mm on average, respectively. Most species in the Order Amphisphaeriales were weak PCL degraders (Table 1).

**Fig. 5:**
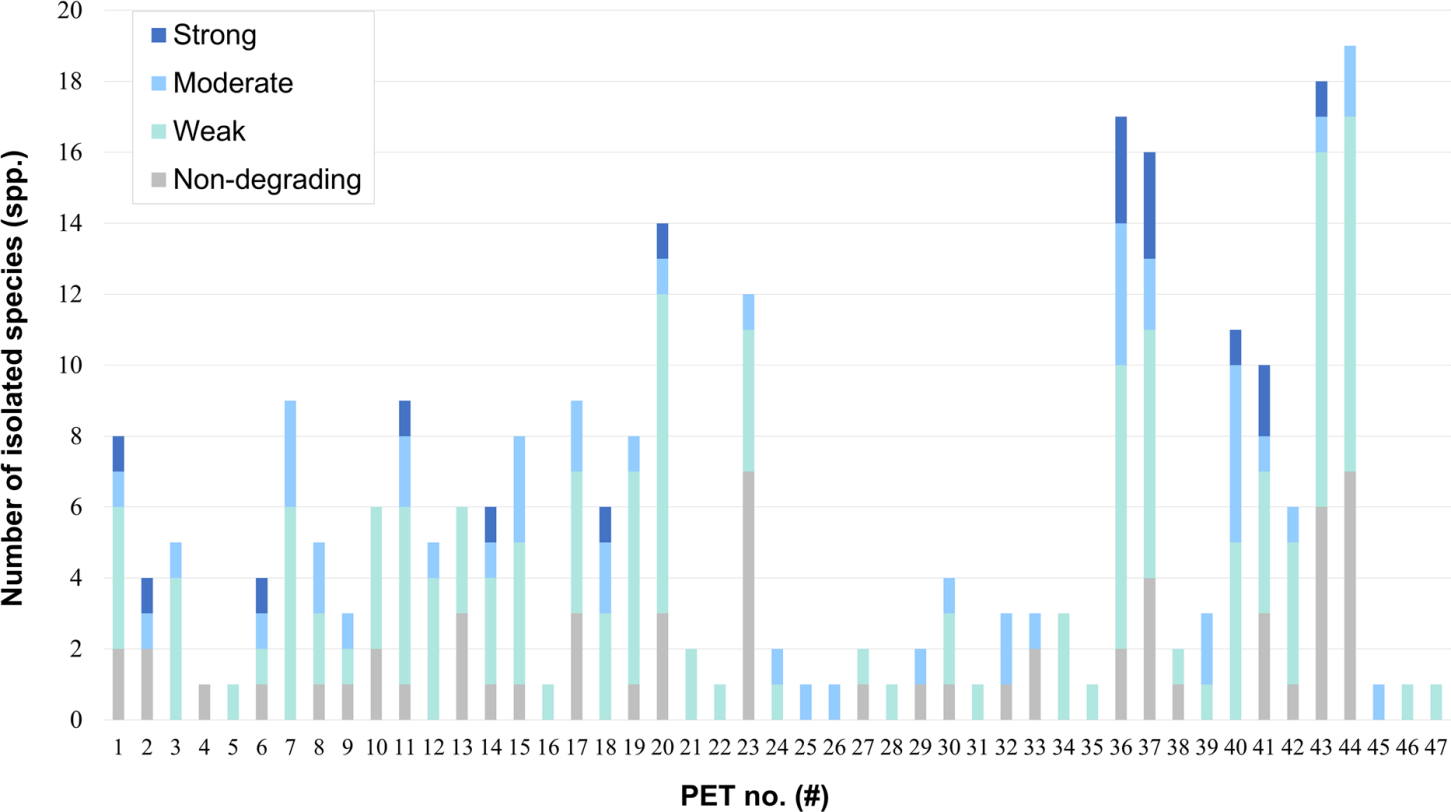
Fungal species isolated from collected plastic wastes. Color intensity is commensurate with PCL degradation level

Whereas most fungi could degrade PCL, certain species isolated from 27 PET waste sources failed to form clear zones on PCL agar. Most of them were isolated along with other PCL-degrading fungi (Fig. 5). Weak and moderate PCL-degrading fungi were detected in most samples. Weakly PCL-degrading fungi were particularly abundant in PET samples Nos. 20, 36, 37, 43, and 44. By contrast, strong PCL degrading fungi did not predominate in any PET samples and always co-occurred either with weak or moderate PCL-degrading fungi or with those that did not degrade PCL at all.

## Discussion

We isolated fungi from marine PET wastes and tested PCL degradation to discover competent plastic-degrading fungi in the marine plastisphere and determined whether they could degrade PET. Numerous fungi were isolated despite the limited number of PET wastes examined here. They resembled that reported in a previous study on fungal diversity in the marine environment (Jones et al. 2015; Kwon et al. 2021). *Acremonium fuci* was isolated from seaweed in Europe and North America (Zuccaro et al. 2004), and several *Cladosporium* species, such as *C. perangustum*, *C. tenuissimum*, and *C. xanthochromaticum* were found in marine sediments (Luo et al., 2020). *Paradendryphiellla arenariae* were reported from various microalgae in Europe (Dela Cruz et al. 2006), and a number of other fungal species including *Fusarium equiseti*, *Nigrospora oryzae*, *Penicillium oxalicum,* and *Trichoderma harzianum* were found in sea sand, mudflats, and seaweeds (Heo et al. 2019; Park et al. 2019). The fungal species detected in this research were also similar to those in other marine and terrestrial plastisphere. Many species in Pleosporales were detected in plastisphere of the Antarctic Ocean (Lacerda et al. 2020) and the North Sea (De Tender et al. 2017). The *Aspergillus*, *Chaetomium*, *Epicoccum, Fusarium,* and *Trichoderma* species were identified in the terrestrial plastisphere (Kemona and Piotrowska 2016; Ye et al., 2021).

Several putative plastic-degrading fungi were identified by the PCL agar degradation test. Approximately 81% of all identified species formed clear zones and were, therefore, potential plastic biodegraders. PET degradation-associated enzyme activity was detected in PCL-degrading fungus (Nyyssölä et al. 2013). Hence, the species identified here could conceivably decompose PET and other plastics as well. *Cladosporium* included 13 PCL-degrading species of which four and nine had strong and moderate PCL-degrading activity, respectively. Prior research confirmed that several *Cladosporium* strains effectively degraded other substrates, such as polyurethane (Bonhomme et al. 2003; Brunner et al. 2018; Srikanth et al. 2022). Therefore, *Cladosporium* species could degrade plastic wastes in the marine environment. *Aureobasidium pullulans*, which displayed relatively good polyurethane degradation were also reported previously (Crabbe et al. 1994). *Phaeophleospora eucalypticola* showed the strongest PCL-degrading activity, but this species has not been given much attention to its degrading abilities. Further research on *P. eucalypticola* may reveal its full potential for the degradation of plastics. It is reported that various enzymes, such as cutinase, laccase, and esterase from fungi were used in degradation of PET (Anbalagan et al. 2021; Khan et al. 2022), and this may explain the high biodiversity in relatively small number of plastic samples.

In this study, most of the highly abundant species showed low levels of PCL degradation ability. *Paradendryphiella arenariae*, Pleosporales sp.1, and *Acremonium* cf. *fuci* were very abundant but relatively weak PCL degraders. In contrast, the fungi with the strongest degradation ability were far less abundant. *Phaeophleospora eucalypticola* and *C. xanthochromaticum* showed strong PCL degradation capacity, but only two strains were isolated from 47 plastic wastes. Each PET waste had many fungal species with low level of PCL degradation but generally possessed only one fungal species that was highly effective. This result allowed us to infer that varying fungal species on plastic waste performed different roles. The abundant weak plastic degraders may grow on plastic wastes to utilize materials primarily degraded by mechanical or biological process. The initial breakdown mechanism of plastics may include polymer oxidation, which increases the hydrophilicity of plastics, weakens their bonds and mechanical structures, and facilitates secondary colonizer access (Oberbeckmann and Labrenz 2020). Microorganisms also gradually degrade other complex molecules such as lignin (Janusz et al. 2017) and anthropogenic (synthetic) polymers (Chen et al. 2020). Hence, the colonizing mechanisms of various microbes strongly influence fungal diversity on plastic wastes. Species with weak or no PCL-degrading ability may have been isolated from the primary colonizers as fungicolous fungi. The inhabitation of fungal species on other plastic-colonizing microorganisms has previously been reported (Webb et al. 2000). Some Hypocreales and Pleosporales species are known to obtain their nutrients either commensally or parasitically from other fungi (Sun et al. 2019). Most species in Hypocreales and Pleosporales showed low PCL degradation ability, proving that they get their nutrients from alternative sources.

In conclusion, the present study showed that numerous fungi inhabit PET wastes in the marine environment. Certain fungal taxa including *Phaeophleospora eucalypticola*, *Alternar*i*a* spp., *Aureobasidium* spp., and *Cladosporium* spp. have strong PCL degrading activity. Fungi with low level of PCL degrading ability were abundant and co-occurred with one of strong PCL degrader. The wide diversity and ranges of abundance, and plastic-degrading capacity of fungi even on small quantities of PET suggest that the various fungal taxa play different roles in marine plastic waste decomposition. In future research, we will aim to clarify the functions of each of these fungal taxa in order to develop a strategy for effective and efficient plastic waste degradation in the marine environment.

## Supplementary Information

Below is the link to the electronic supplementary material.

Supplementary file 1 Table S1: Collected PET wastes and isolated fungal species. Representative strains and cultures whence fungal species were isolated are also indicated. Table S2: Strains and their GenBank accession numbers used for phylogenetic analyses in this study. Table S3: Clear zone data for all tested fungal strains. The degradation abilities are categorized into four level

## References

[CR1] Ahrendt C, Perez-Venegas DJ, Urbina M (2020). Microplastic ingestion cause intestinal lesions in the intertidal fish *Girella laevifrons*. Mar Pollut Bull.

[CR2] Amaral-Zettler LA, Zettler ER, Mincer TJ (2020). Ecology of the plastisphere. Nat Rev Microbiol.

[CR3] Anbalagan S, Venkatakrishnan HRR, Ravindran J (2021). Hydrolytic degradation of polyethylene terephthalate by cutinase enzyme derived from fungal biomass–molecular characterization. BioInterface Res Appl Chem..

[CR4] Arias-Andres M, Klümper U, Rojas-Jimenez K, Grossart HP (2018). Microplastic pollution increases gene exchange in aquatic ecosystems. Environ Pollut.

[CR5] Aveskamp MM, Woudenberg JH, De Gruyter J, Turco E, Groenewald JZ, Crous PW (2009). Development of taxon-specific sequence characterized amplified region (SCAR) markers based on actin sequences and DNA amplification fingerprinting (DAF): a case study in the *Phoma exigua* species complex. Mol Plant Pathol.

[CR6] Badahit G, Kumar J, Singh A (2018). Screening of plastic degrading *Pseudomonas* spp. from soil. Int J Sci Eng Res.

[CR7] Beloe CJ, Browne MA, Johnston EL (2022). Plastic debris as a vector for bacterial disease: An interdisciplinary systematic review. Environ Sci Technol.

[CR8] Benedict CV, Cook WJ, Jarrett P, Cameron JA, Huang SJ, Bell JP (1983). Fungal degradation of polycaprolactones. J Appl Polym Sci.

[CR9] Bonhomme S, Cuer A, Delort AM, Lemaire J, Sancelme M, Scott G (2003). Environmental biodegradation of polyethylene. Polym Degrad.

[CR10] Bowley J, Baker-Austin C, Porter A, Hartnell R, Lewis C (2021). Oceanic hitchhikers–assessing pathogen risks from marine microplastic. Trends Microbiol.

[CR11] Brunner I, Fischer M, Rüthi J, Stierli B, Frey B (2018). Ability of fungi isolated from plastic debris floating in the shoreline of a lake to degrade plastics. PLoS ONE.

[CR12] Carbone I, Kohn LM (1999). A method for designing primer sets for speciation studies in filamentous ascomycetes. Mycologia.

[CR13] Chen CC, Dai L, Ma L, Guo RT (2020). Enzymatic degradation of plant biomass and synthetic polymers. Nat Rev Chem.

[CR14] Cosgrove L, McGeechan PL, Robson GD, Handley PS (2007). Fungal communities associated with degradation of polyester polyurethane in soil. Appl Environ Microbiol.

[CR15] Crabbe JR, Campbell JR, Thompson L, Walz SL, Schultz WW (1994). Biodegradation of a colloidal ester-based polyurethane by soil fungi. Int Biodeterior Biodegrad.

[CR16] Davidov K, Iankelevich-Kounio E, Yakovenko I, Koucherov Y, Rubin-Blum M, Oren M (2020). Identification of plastic-associated species in the Mediterranean Sea using DNA metabarcoding with nanopore MinION. Sci Rep.

[CR17] De Tender C, Devriese LI, Haegeman A, Maes S, Vangeyte J, Cattrijsse A, Dawyndt P, Ruttink T (2017). Temporal dynamics of bacterial and fungal colonization on plastic debris in the North Sea. Environ Sci Technol.

[CR18] Dela Cruz TEE, Schulz BE, Kubicek CP, Druzhinina IS (2006). Carbon source utilization by the marine *Dendryphiella* species *D. arenaria* and *D. salina*. FEMS Microbiol Ecol.

[CR19] Fukushima K, Abbate C, Tabuani D, Gennari M, Rizzarelli P, Camino G (2010). Biodegradation trend of poly (ε-caprolactone) and nanocomposites. Mater Sci Eng C.

[CR20] Gardes M, Bruns TD (1993). ITS primers with enhanced specificity for basidiomycetes–application to the identification of mycorrhizae and rusts. Mol Ecol.

[CR21] Geyer R, Jambeck JR, Law KL (2017). Production, use, and fate of all plastics ever made. Sci Adv.

[CR22] Glass NL, Donaldson GC (1995). Development of primer sets designed for use with the PCR to amplify conserved genes from filamentous ascomycetes. Appl Environ Microbiol.

[CR23] Groenewald JZ, Nakashima C, Nishikawa J (2013). Species concepts in *Cercospora*: spotting the weeds among the roses. Stud Mycol.

[CR24] Heo YM, Lee H, Kim K (2019). Fungal diversity in intertidal mudflats and abandoned solar salterns as a source for biological resources. Mar Drugs.

[CR25] Hirota Y, Naya M, Tada M, Shikyo Y, Kawanishi T, Takiguchi N (2021). Analysis of soil fungal community structure on the surface of buried polyethylene terephthalate. J Polym Environ.

[CR26] Hou L, Xi J, Liu J, Wang P, Xu T, Liu T, Qu W, Lin YB (2022). Biodegradability of polyethylene mulching film by two *Pseudomonas* bacteria and their potential degradation mechanism. Chemosphere.

[CR27] Jambeck JR, Geyer R, Wilcox C, Siegler TR, Perryman M, Andrady A, Narayan R, Law KL (2015). Marine pollution. Plastic waste inputs from land into the ocean. Science.

[CR28] Janusz G, Pawlik A, Sulej J, Świderska-Burek U, Jarosz-Wilkołazka A, Paszczyński A (2017). Lignin degradation: microorganisms, enzymes involved, genomes analysis and evolution. FEMS Microbiol Rev.

[CR29] Jones EB, Suetrong S, Sakayaroj J, Bahkali AH, Abdel-Wahab MA, Boekhout T, Pang KL (2015). Classification of marine Ascomycota, Basidiomycota, Blastocladiomycota and Chytridiomycota. Fungal Divers.

[CR30] Kanelli M, Vasilakos S, Nikolaivits E, Ladas S, Christakopoulos P, Topakas E (2015). Surface modification of poly (ethylene terephthalate) (PET) fibers by a cutinase from *Fusarium oxysporum*. Process Biochem.

[CR31] Katoh K, Standley DM (2013). MAFFT multiple sequence alignment software 7: improvements in performance and usability. Mol Biol Evol.

[CR32] Kemona A, Piotrowska M (2016). Microorganisms potentially useful in the management of polyurethane foam waste. Infrastruct Ecol Rural Area.

[CR33] Khan S, Ali SA, Ali AS (2022). Biodegradation of low-density polyethylene (LDPE) by mesophilic fungus ‘*Penicillium citrinum*’ isolated from soils of plastic waste dump yard, Bhopal. India Environ Technol.

[CR34] Kumar S, Stecher G, Tamura K (2016). MEGA7: molecular evolutionary genetics analysis version 7.0 for bigger datasets. Mol Biol Evol.

[CR35] Kumari A, Chaudhary DR, Jha B (2019). Destabilization of polyethylene and polyvinylchloride structure by marine bacterial strain. Environ Sci Pollut Res Int.

[CR36] Kwon YM, Bae SS, Choi G, Lim JY, Jung YH, Chung D (2021). Marine-derived fungi in Korea. Ocean Sci J.

[CR37] Lacerda ALDF, Proietti MC, Secchi ER, Taylor JD (2020). Diverse groups of fungi are associated with plastics in the surface waters of the Western South Atlantic and the Antarctic Peninsula. Mol Ecol.

[CR38] Lebreton LCM, Van Der Zwet J, Damsteeg JW, Slat B, Andrady A, Reisser J (2017). River plastic emissions to the world’s oceans. Nat Comm.

[CR39] Lee S, Park MS, Lee H, Kim JJ, Eimes JA, Lim YW (2019). Fungal diversity and enzyme activity associated with the macroalgae, *Agarum clathratum*. Mycobiology.

[CR40] Lee SY, Ten LN, Das K, You YH, Jung HY (2021). Biodegradative activities of fungal strains isolated from terrestrial environments in Korea. Mycobiology.

[CR41] Luo Y, Xu W, Luo ZH, Pang KL (2020). Diversity and temperature adaptability of cultivable fungi in marine sediments from the Chukchi Sea. Bot Mar.

[CR42] Muhonja CN, Makonde H, Magoma G, Imbuga M (2018). Biodegradability of polyethylene by bacteria and fungi from Dandora dumpsite Nairobi-Kenya. PLoS ONE.

[CR43] Nyyssölä A, Pihlajaniemi V, Järvinen R, Mikander S, Kontkanen H, Kruus K, Kallio H, Buchert J (2013). Screening of microbes for novel acidic cutinases and cloning and expression of an acidic cutinase from *Aspergillus niger* CBS 513.88. Enzyme Microb Technol.

[CR44] Oberbeckmann S, Labrenz M (2020). Marine microbial assemblages on microplastics: diversity, adaptation, and role in degradation. Annu Rev Mar Sci.

[CR45] Paço A, Duarte K, da Costa JP (2017). Biodegradation of polyethylene microplastics by the marine fungus *Zalerion maritimum*. Sci Total Environ.

[CR46] Park MS, Eom JE, Fong JJ, Lim YW (2015). New record and enzyme activity of four species in *Penicillium* section *Citrina* from marine environments in Korea. J Microbiol.

[CR47] Park MS, Fong JJ, Oh SY, Houbraken J, Sohn JH, Hong SB, Lim YW (2015). *Penicillium jejuense* sp. nov., isolated from the marine environments of Jeju Island. Korea Mycologia.

[CR48] Park MS, Lee S, Oh SY, Cho GY, Lim YW (2016). Diversity and enzyme activity of *Penicillium* species associated with macroalgae in Jeju Island. J Microbiol.

[CR49] Park MS, Oh SY, Lee S, Eimes JA, Lim YW (2018). Fungal diversity and enzyme activity associated with sailfin sandfish egg masses in Korea. Fungal Ecol.

[CR50] Park MS, Oh SY, Fong JJ, Houbraken J, Lim YW (2019). The diversity and ecological roles of *Penicillium* in intertidal zones. Sci Rep.

[CR51] Plastics Europe (2021) Plastics-the Facts 2021. https://plasticseurope.org/knowledge-hub/plastics-the-facts-2021/*.* Accessed 25 February 2022

[CR52] Rios LM, Moore C, Jones PR (2007). Persistent organic pollutants carried by synthetic polymers in the ocean environment. Mar Pollut Bull.

[CR53] Rogers SO, Bendich AJ, Gelvin S, Schilperoort RA (1994). Extraction of total cellular DNA from plants, algae and fungi. Plant molecular biology manual.

[CR54] Rogers KL, Carreres-Calabuig JA, Gorokhova E, Posth NR (2020). Micro-by-micro interactions: How microorganisms influence the fate of marine microplastics. Limnol Oceanogr Lett.

[CR55] Sangeetha Devi R, Ramya R, Kannan K, Robert Antony A, Rajesh Kannan V (2019). Investigation of biodegradation potentials of high-density polyethylene degrading marine bacteria isolated from the coastal regions of Tamil Nadu, India. Mar Pollut Bull.

[CR56] Sarkhel R, Sengupta S, Das P, Bhowal A (2020). Comparative biodegradation study of polymer from plastic bottle waste using novel isolated bacteria and fungi from marine source. J Polym Res.

[CR57] Srikanth M, Sandeep TSRS, Sucharitha K, Godi S (2022). Biodegradation of plastic polymers by fungi: A brief review. Bioresour Bioprocess.

[CR58] Stamatakis A (2006). RAxML-VI-HPC: maximum likelihood-based phylogenetic analyses with thousands of taxa and mixed models. Bioinform.

[CR59] Sun JZ, Liu XZ, McKenzie EH, Jeewon R, Liu ZX, Zhao Q, Hyde KD (2019). Fungicolous fungi: terminology, diversity, distribution, evolution, and species checklist. Fungal Divers.

[CR60] Temporiti MEE, Nicola L, Nielsen E, Tosi S (2022). Fungal enzymes involved in plastics biodegradation. Microorganisms.

[CR61] Thompson RC, Olsen Y, Mitchell RP (2004). Lost at sea: where is all the plastic?. Science.

[CR62] Webb JS, Nixon M, Eastwood IM, Greenhalgh M, Robson GD, Handley PS (2000). Fungal colonization and biodeterioration of plasticized polyvinyl chloride. Appl Environ Microbiol.

[CR63] White TJ, Bruns T, Lee S, Taylor J, Innis MA, Gelfand H, Sninsky JJ, White TJ (1990). Amplification and direct sequencing of fungal ribosomal RNA genes for phylogenetics. PCR protocols: a guide to methods and applications.

[CR64] Wright SL, Rowe D, Thompson RC, Galloway TS (2013). Microplastic ingestion decreases energy reserves in marine worms. Curr Biol.

[CR65] Yamada-Onodera K, Mukumoto H, Katsuyaya Y, Saiganji A, Tani Y (2001). Degradation of polyethylene by a fungus, *Penicillium simplicissimum* YK. Polym Degrad Stabil.

[CR66] Ye R, Xu S, Wang Q, Fu X, Dai H, Lu W (2021). Fungal diversity and its mechanism of community shaping in the milieu of sanitary landfill. Front Environ Sci Eng.

[CR67] Zahra S, Abbas SS, Mahsa MT, Mohsen N (2010). Biodegradation of low-density polyethylene (LDPE) by isolated fungi in the solid waste medium. Waste Manag.

[CR68] Zettler ER, Mincer TJ, Amaral-Zettler LA (2013). Life in the “plastisphere”: microbial communities on plastic marine debris. ES&T.

[CR69] Zuccaro A, Summerbell RC, Gams W, Schroers HJ, Mitchell JI (2004). A new *Acremonium* species associated with *Fucus* spp., and its affinity with a phylogenetically distinct marine *Emericellopsis* clade. Stud Mycol.

