# Supplementary material for: Plastic-inhabiting fungi in marine environments and PCL degradation activity
Source: Antonie Van Leeuwenhoek. 2022 Oct 14;115(12):1379–92. doi: 10.1007/s10482-022-01782-0 (PMC9675664; doi:10.1007/s10482-022-01782-0)
Supplement: Supplementary file 1 — Table S1: Collected PET wastes and isolated fungal species. Representative strains and cultures whence fungal species were isolated are also indicated. Table S2: Strains and their GenBank accession numbers used for phylogenetic analyses in this study. Table S3: Clear zone data for all tested fungal strains. The degradation abilities are categorized into four level [file 10482_2022_1782_MOESM1_ESM.docx]

# Plastic-Inhabiting Fungi in Marine Environments and PCL Degradation Analysis

Antoine van Leeuwenhoek

**Sung Hyun Kim^1^**, Jun Won Lee^1^, Ji Seon Kim^1^, Wonjun Lee^1^, Myung Soo Park^1,2^, and Young Woon Lim^1^*

^1^ School of Biological Sciences and Institution of Microbiology, Seoul National University, Seoul 08826, Republic of Korea

^2^ Department of Crops and Forestry, Korea National College of Agriculture and Fisheries, Jeonju 54874, Republic of Korea

^*^Corresponding author: [ywlim@snu.ac.kr](mailto:ywlim@snu.ac.kr)

**Supplementary Figure S1. Phylogenetic trees based on ML analysis of actin (*act*) for *Cladosporium* (A), β-tubulin (*BenA*) for *Diaporthe* (B), *Didymella*/*Epicoccum /Juxtiphoma*/*Neodidymelliopsis*/*Nothophoma*/*Remotididymella* (Didymellaceae) (C), *Aspergillus*/*Penicillium*/*Talaromyces*(D), and *Pestalotiopsis*/*Neopestalotiopsis* (E). Bootstrap scores are presented at the nodes if > 70.**


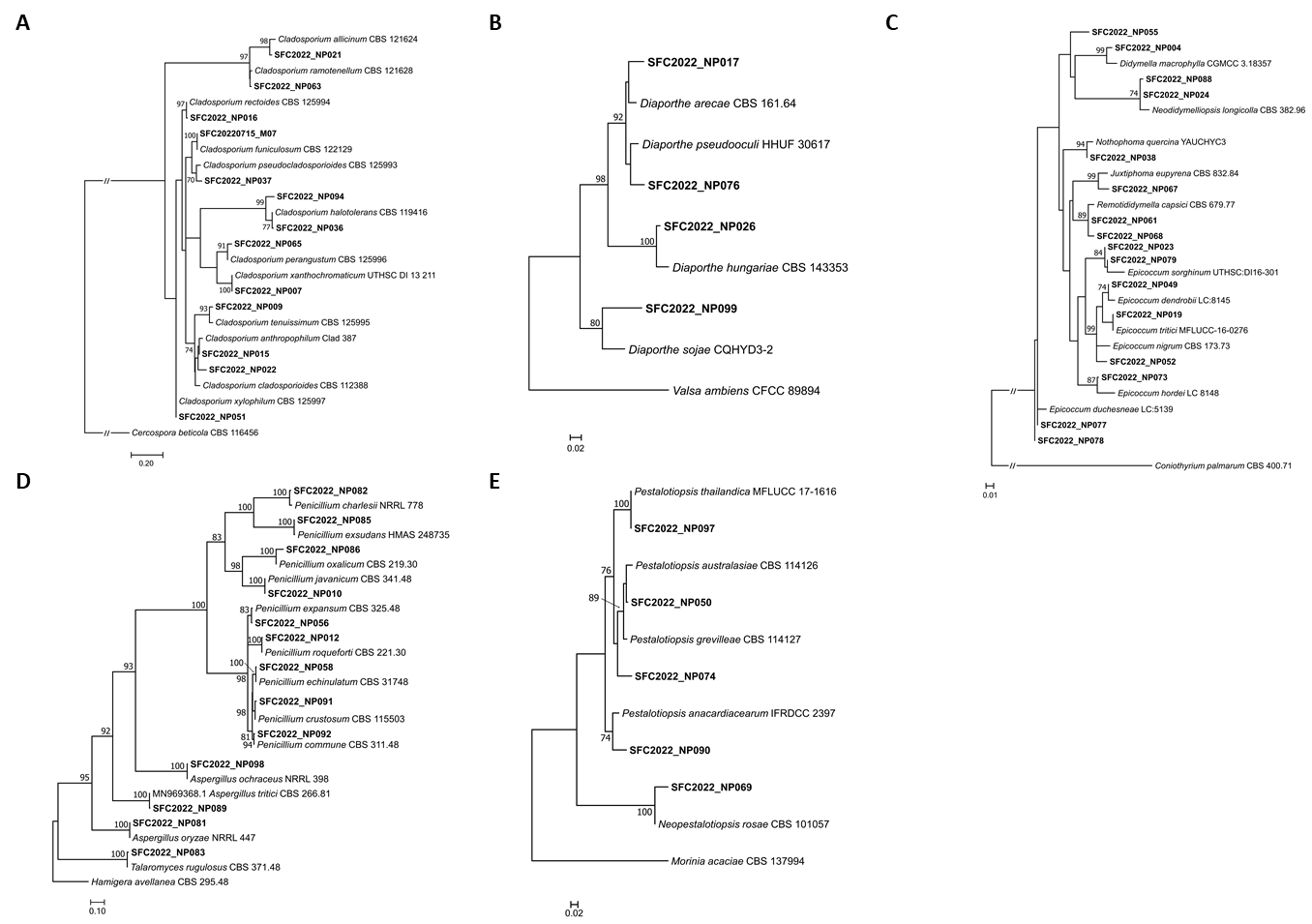


**Supplementary Table S1. Collected PET wastes and isolated fungal species.** Representative strains and cultures whence fungal species were isolated are also indicated.

| **Site** | **Mud/Sand** | **Location** | **Coordinates**  **(Latitude, Longitude)** | **PET code** | **Species name** | **Culture** |
| --- | --- | --- | --- | --- | --- | --- |
| Site 1 | Sand | Gwanpo-ri, Jangmok-myeon, Geoje-si, Gyeongsangnam-do | 34.9904, 128.6950 | 1 | *Acremonium* cf. *fuci* | PDA |
|  |  |  |  |  | *Acremonium fuci* | PDA |
|  |  |  |  |  | *Alternaria alternata* | GYA |
|  |  |  |  |  | *Cladosporium ramotenellum* | GYA/PDA |
|  |  |  |  |  | *Cladosporium tenuissimum* | DRBC |
|  |  |  |  |  | *Cladosporium xylophilum* | PDA |
|  |  |  |  |  | *Neodidymelliopsis* cf. *longicolla* | DRBC |
|  |  |  |  |  | *Paradendryphiella arenariae* | DRBC |
|  |  |  |  | 2 | *Acremonium* cf. *fuci* | GYA |
|  |  |  |  |  | *Acremonium fuci* | GYA |
|  |  |  |  |  | *Cladosporium anthropophilum* | GYA |
|  |  |  |  |  | *Cladosporium rectoides* | GYA |
|  |  |  |  | 3 | *Alternaria alternata* | GYA/PDA |
|  |  |  |  |  | *Alternaria chlamydospora* | PDA |
|  |  |  |  |  | *Cladosporium ramotenellum* | PDA |
|  |  |  |  |  | *Paradendryphiella arenariae* | GYA |
|  |  |  |  |  | *Stemphylium vesicarium* | GYA |
|  |  |  |  | 4 | Pleosporales sp. 1 | GYA |
|  |  |  |  | 5 | Pleosporaceae sp. 2 | DRBC |
|  |  |  |  | 6 | *Alternaria alternata* | GYA |
|  |  |  |  |  | *Cladosporium ramotenellum* | GYA |
|  |  |  |  |  | *Cladosporium tenuissimum* | GYA |
|  |  |  |  |  | Pleosporales sp. 1 | PDA |
|  |  |  |  | 7 | *Alternaria alternata* | DRBC/GYA/PDA |
|  |  |  |  |  | *Cladosporium pseudocladosporioides* | PDA |
|  |  |  |  |  | *Cladosporium ramotenellum* | GYA/PDA |
|  |  |  |  |  | *Diaporthe* cf. *hungariae* | PDA |
|  |  |  |  |  | *Epicoccum dendrobii* | PDA |
|  |  |  |  |  | *Epicoccum* sp. | PDA |
|  |  |  |  |  | *Parasarocladium* cf. *gamsii* | GYA |
|  |  |  |  |  | *Pestalotiopsis* cf. *australasiae* | DRBC |
|  |  |  |  |  | *Septoriella* cf. *hubertusii* | GYA/PDA |
|  |  |  |  | 8 | *Alternaria alternata* | PDA |
|  |  |  |  |  | *Cladosporium anthropophilum* | PDA |
|  |  |  |  |  | *Cladosporium ramotenellum* | PDA |
|  |  |  |  |  | Pleosporales sp. 1 | PDA |
|  |  |  |  |  | Pleosporales sp. 2 | PDA |
|  |  |  |  | 9 | *Alternaria alternata* | GYA/PDA |
|  |  |  |  |  | *Cladosporium ramotenellum* | PDA |
|  |  |  |  |  | Pleosporales sp. 1 | PDA |
|  |  |  |  | 10 | *Cladosporium ramotenellum* | GYA/PDA |
|  |  |  |  |  | *Cladosporium xylophilum* | PDA |
|  |  |  |  |  | *Fusarium equiseti* | DRBC/GYA |
|  |  |  |  |  | *Neocamarosporium betae* | PDA |
|  |  |  |  |  | *Paradendryphiella arenariae* | DRBC/PDA |
|  |  |  |  |  | Pleosporales sp. 1 | PDA |
| Site 2 | Mud | Jangmok-ri, Jangmok-myeon, Geoje-si, Gyeongsangnam-do | 34.9916, 128.6810 | 11 | *Acremonium fuci* | GYA |
|  |  |  |  |  | *Alternaria alternata* | GYA/PDA |
|  |  |  |  |  | *Apiospora rasikravindrae* | PDA |
|  |  |  |  |  | *Aureobasidium pullulans* | GYA |
|  |  |  |  |  | *Cladosporium anthropophilum* | PDA |
|  |  |  |  |  | *Cladosporium funiculosum* | GYA |
|  |  |  |  |  | *Cladosporium ramotenellum* | GYA/PDA |
|  |  |  |  |  | *Cladosporium tenuissimum* | PDA |
|  |  |  |  |  | Didymosphaeriaceae sp. *2* | GYA |
|  |  |  |  | 12 | *Cladosporium ramotenellum* | DRBC |
|  |  |  |  |  | *Cladosporium rectoides* | DRBC |
|  |  |  |  |  | *Cladosporium xylophilum* | PDA |
|  |  |  |  |  | *Diaporthe* cf. *hungariae* | DRBC |
|  |  |  |  |  | *Neodidymelliopsis* cf. *longicolla* | DRBC |
|  |  |  |  | 13 | *Acremonium* cf. *fuci* | GYA |
|  |  |  |  |  | *Acremonium fuci* | GYA |
|  |  |  |  |  | *Cladosporium ramotenellum* | GYA |
|  |  |  |  |  | *Diaporthe* cf. *hungariae* | DRBC/PDA |
|  |  |  |  |  | *Paraconiothyrium brasiliense* | DRBC |
|  |  |  |  |  | *Paradendryphiella arenariae* | DRBC/GYA |
| Site 3 | Mud | Dongdal-ri, Yongnam-myeon, Tongyeong-si, Gyeongsangnam-do | 34.8701, 128.4551 | 14 | *Acremonium* cf. *fuci* | GYA |
|  |  |  |  |  | *Cladosporium ramotenellum* | GYA/PDA |
|  |  |  |  |  | *Cladosporium xanthochromaticum* | GYA |
|  |  |  |  |  | *Epicoccum dendrobii* | PDA |
|  |  |  |  |  | *Paradendryphiella arenariae* | PDA |
|  |  |  |  |  | *Parasarocladium* cf. *gamsii* | PDA |
|  |  |  |  | 15 | *Acremonium* cf. *fuci* | GYA |
|  |  |  |  |  | *Alternaria alternata* | DRBC |
|  |  |  |  |  | *Cladosporium anthropophilum* | PDA |
|  |  |  |  |  | *Fusarium equiseti* | DRBC |
|  |  |  |  |  | *Neodidymelliopsis longicolla* | GYA |
|  |  |  |  |  | *Paradendryphiella arenariae* | DRBC/GYA |
|  |  |  |  |  | *Remotididymella* cf. *capsici* | DRBC/PDA |
|  |  |  |  |  | *Sphaeropsis sapinea* | GYA |
|  |  |  |  | 16 | *Paradendryphiella arenariae* | GYA |
| Site 4 | Sand | Wonpyeong-ri, Yongnam-myeon, Tongyeong-si, Gyeongsangnam-do | 34.9005, 128.4561 | 17 | *Acremonium* cf. *fuci* | PDA |
|  |  |  |  |  | *Acremonium fuci* | GYA |
|  |  |  |  |  | *Alternaria alternata* | GYA |
|  |  |  |  |  | *Cladosporium ramotenellum* | GYA/PDA |
|  |  |  |  |  | *Epicoccum duchesneae* | PDA |
|  |  |  |  |  | *Neodevriesia* cf. *metrosideri* | PDA |
|  |  |  |  |  | *Neodidymelliopsis* cf. *longicolla* | GYA |
|  |  |  |  |  | *Paradendryphiella arenariae* | DRBC/GYA/PDA |
|  |  |  |  |  | Pleosporales sp. 1 | GYA |
|  |  |  |  | 18 | *Alternaria alternata* | GYA |
|  |  |  |  |  | *Cladosporium allicinum* | GYA |
|  |  |  |  |  | *Cladosporium anthropophilum* | GYA |
|  |  |  |  |  | *Diaporthe* cf. *arecae* | GYA |
|  |  |  |  |  | *Fusarium equiseti* | GYA |
|  |  |  |  |  | *Paradendryphiella arenariae* | GYA |
|  |  |  |  | 19 | *Acremonium* cf. *fuci* | GYA |
|  |  |  |  |  | *Alternaria alternata* | PDA |
|  |  |  |  |  | *Cladosporium ramotenellum* | PDA |
|  |  |  |  |  | *Epicoccum tritici* | GYA |
|  |  |  |  |  | *Fusarium fujikuroi* | PDA |
|  |  |  |  |  | *Neodidymelliopsis* cf. *longicolla* | PDA |
|  |  |  |  |  | *Paradendryphiella arenariae* | GYA/PDA |
|  |  |  |  |  | *Remotididymella* cf. *capsici* | PDA |
|  |  |  |  | 20 | *Acremonium* cf. *fuci* | PDA |
|  |  |  |  |  | *Acremonium fuci* | GYA |
|  |  |  |  |  | *Aureobasidium pullulans* | DRBC |
|  |  |  |  |  | *Cladosporium perangustum* | GYA |
|  |  |  |  |  | *Cladosporium tenuissimum* | GYA |
|  |  |  |  |  | *Cladosporium xylophilum* | PDA |
|  |  |  |  |  | *Diaporthe* cf. *hungariae* | PDA |
|  |  |  |  |  | *Fusarium equiseti* | PDA |
|  |  |  |  |  | *Neosetophoma poaceicola* | GYA |
|  |  |  |  |  | *Paradendryphiella arenariae* | DRBC/GYA/PDA |
|  |  |  |  |  | Pleosporales sp. 1 | PDA |
|  |  |  |  |  | *Remotididymella* cf. *capsici* | DRBC/PDA |
|  |  |  |  |  | *Stemphylium lycopersici* | DRBC |
|  |  |  |  |  | *Trichoderma harzianum* | DRBC |
| Site 5 | Mud | Jangpyeong-ri, Yongnam-myeon, Tongyeong-si, Gyeongsangnam-do | 34.8808, 128.4689 | 21 | *Alternaria chlamydospora* | PDA |
|  |  |  |  |  | *Cladosporium ramotenellum* | PDA |
|  |  |  |  | 22 | *Cladosporium ramotenellum* | PDA |
|  |  |  |  | 23 | *Acremonium* cf. *fuci* | PDA |
|  |  |  |  |  | *Alternaria alternata* | PDA |
|  |  |  |  |  | *Alternaria chlamydospora* | PDA |
|  |  |  |  |  | *Cladosporium ramotenellum* | PDA |
|  |  |  |  |  | *Remotididymella* sp. | PDA |
|  |  |  |  |  | *Juxtiphoma* cf. *eupyrena* | PDA |
|  |  |  |  |  | *Neocamarosporium betae* | PDA |
|  |  |  |  |  | *Penicillium commune* | PDA |
|  |  |  |  |  | *Penicillium crustosum* | PDA |
|  |  |  |  |  | *Penicillium echinulatum* | PDA |
|  |  |  |  |  | *Penicillium expansum* | PDA |
|  |  |  |  |  | Pleosporales sp. 1 | PDA |
|  |  |  |  | 24 | *Alternaria alternata* | GYA |
|  |  |  |  |  | *Paradendryphiella arenariae* | GYA |
| Site 6 | Sand | Samsung-ri, Ilgwang-myeon, Gijang-gun, Busan | 35.2632, 129.2353 | 25 | *Alternaria alternata* | PDA |
|  |  |  |  | 26 | *Alternaria alternata* | GYA |
| Site 7 | Mud | Dadae-dong, Saha-gu, Busan | 35.0473, 128.9721 | 27 | *Acremonium* cf. *fuci* | GYA |
|  |  |  |  |  | *Paradendryphiella arenariae* | GYA |
| Site 8 | Mud | Masan-ri, Hyeongyeong-myeon, Muan-gun, Jeollanam-do | 35.0654, 126.3423 | 28 | *Neocamarosporium solicola* | DRBC |
| Site 9 | Mud | Songjeong-ri, Hyeongyeong-myeon, Muan-gun, Jeollanam-do | 35.0317, 126.3906 | 29 | *Chaetomium globosum* | GYA |
|  |  |  |  |  | *Phaeosphaeria spartinicola* | GYA |
| Site 10 | Mud | Jangyang-ri, Beolgyo-eup, Boseong-gun, Jeollanam-do | 34.8364, 127.3694 | 30 | *Alternaria alternata* | DRBC |
|  |  |  |  |  | Pleosporales sp. 1 | DRBC/GYA |
|  |  |  |  |  | Pleosporales sp. 2 | GYA |
|  |  |  |  |  | Pleosporales sp. 4 | DRBC/GYA |
|  |  |  |  | 31 | *Cladosporium halotolerans* | PDA |
| Site 11 | Mud | Masan-ri, Byeollyang-myeon, Suncheon-si, Jeollanam-do | 34.8372, 127.4476 | 32 | *Alternaria alternata* | DRBC |
|  |  |  |  |  | *Cladosporium anthropophilum* | DRBC |
|  |  |  |  |  | *Nigrospora* cf. *oryzae* | GYA |
|  |  |  |  | 33 | *Eutypella* cf. *persica* | PDA |
|  |  |  |  |  | *Nigrospora* cf. *oryzae* | DRBC |
|  |  |  |  |  | *Cytospora ceratosperma* | PDA |
|  |  |  |  | 34 | *Plectosphaerella cucumerina* | GYA |
|  |  |  |  |  | Pleosporales sp. 4 | PDA |
|  |  |  |  |  | *Sedecimiella taiwanensis* | DRBC |
|  |  |  |  | 35 | *Trichoderma fomiticola* | GYA |
| Site 12 | Mud | Boksan-ri, Sora-myeon, Yeosu-si, Jeollanam-do | 34.7735, 127.5725 | 36 | *Acremonium* cf. *fuci* | PDA |
|  |  |  |  |  | *Alternaria alternata* | DRBC/GYA/PDA |
|  |  |  |  |  | *Apiospora marii* | GYA |
|  |  |  |  |  | *Botrytis cinerea* | PDA |
|  |  |  |  |  | *Cladosporium allicinum* | GYA |
|  |  |  |  |  | *Cladosporium anthropophilum* | DRBC/GYA |
|  |  |  |  |  | *Cladosporium* cf. *cladosporioides* | GYA |
|  |  |  |  |  | *Cladosporium pseudocladosporioides* | GYA |
|  |  |  |  |  | *Cladosporium rectoides* | DRBC |
|  |  |  |  |  | *Cladosporium tenuissimum* | PDA |
|  |  |  |  |  | *Diaporthe* cf. *hungariae* | DRBC |
|  |  |  |  |  | *Didymella* cf. *macrophylla* | DRBC |
|  |  |  |  |  | *Epicoccum tritici* | DRBC/PDA |
|  |  |  |  |  | *Neosetophoma* cf. *poaceicola* | DRBC |
|  |  |  |  |  | *Penicillium javanicum* | PDA |
|  |  |  |  |  | *Penicillium roqueforti* | PDA |
|  |  |  |  |  | *Pyrenochaetopsis paucisetosa* | DRBC |
|  |  |  |  | 37 | *Stemphylium vesicarium* | PDA |
|  |  |  |  |  | *Acremonium* cf. *fuci* | DRBC/GYA |
|  |  |  |  |  | *Alternaria alternata* | DRBC |
|  |  |  |  |  | *Cladosporium perangustum* | DRBC |
|  |  |  |  |  | *Cladosporium rectoides* | DRBC |
|  |  |  |  |  | *Cladosporium tenuissimum* | DRBC |
|  |  |  |  |  | *Cladosporium xanthochromaticum* | DRBC |
|  |  |  |  |  | *Diaporthe* cf. *arecae* | GYA |
|  |  |  |  |  | *Epicoccum* cf. *sorghinum* | DRBC |
|  |  |  |  |  | *Epicoccum sorghinum* | DRBC |
|  |  |  |  |  | *Neocamarosporium betae* | DRBC/GYA/PDA |
|  |  |  |  |  | *Neocamarosporium* sp. | DRBC |
|  |  |  |  |  | *Neodidymelliopsis longicolla* | DRBC |
|  |  |  |  |  | *Paradendryphiella arenariae* | DRBC/GYA |
|  |  |  |  |  | *Phaeosphaeria culmorum* | PDA |
|  |  |  |  |  | Pleosporales sp. 1 | GYA |
| Site 13 | Mud | Jinseo-ri, Jinseo-myeon, Buan-gun, Jeollabuk-do | 35.5950, 126.6033 | 38 | Hypocreales sp. | DRBC |
|  |  |  |  |  | *Parengyodontium album* | GYA |
|  |  |  |  | 39 | *Alternaria alternata* | GYA |
|  |  |  |  |  | *Alternaria* cf. *rosae* | GYA |
|  |  |  |  |  | *Neocamarosporium* sp. | DRBC/GYA/PDA |
|  |  |  |  | 40 | *Alternaria alternata* | PDA |
|  |  |  |  |  | *Alternaria* cf. *rosae* | DRBC/PDA |
|  |  |  |  |  | *Aureobasidium melanogenum* | DRBC/PDA |
|  |  |  |  |  | *Aureobasidium namibiae* | DRBC |
|  |  |  |  |  | *Cladosporium pseudocladosporioides* | DRBC |
|  |  |  |  |  | *Cladosporium rectoides* | GYA |
|  |  |  |  |  | *Neocamarosporium solicola* | DRBC |
|  |  |  |  |  | *Neocamarosporium* sp. | GYA |
|  |  |  |  |  | *Neodidymelliopsis* cf. *longicolla* | PDA |
|  |  |  |  |  | *Neosetophoma rosigena* | DRBC/PDA |
|  |  |  |  |  | *Parathyridaria* cf. *tyrrhenica* | GYA |
| Site 14 | Sand | Jungmun-dong, Seogwipo-si, Jeju-do | 33.2427, 126.4188 | 41 | *Acremonium fuci* | DRBC |
|  |  |  |  |  | *Aspergillus ochraceus* | GYA/PDA |
|  |  |  |  |  | *Cladosporium tenuissimum* | GYA |
|  |  |  |  |  | *Diaporthe* cf. *pseudooculi* | GYA |
|  |  |  |  |  | *Didymosphaeriaceae* sp. 1 | DRBC |
|  |  |  |  |  | *Epicoccum* cf. *duchesneae* | GYA |
|  |  |  |  |  | *Parathyridaria* cf. *tyrrhenica* | GYA |
|  |  |  |  |  | *Penicillium charlesii* | PDA |
|  |  |  |  |  | *Penicillium crustosum* | DRBC |
|  |  |  |  |  | *Phaeophleospora eucalypticola* | GYA |
|  |  |  |  | 42 | *Cladosporium* cf. *halotolerans* | PDA |
|  |  |  |  |  | *Epicoccum* cf. *duchesneae* | PDA |
|  |  |  |  |  | *Morinia* cf. *acaciae* | GYA |
|  |  |  |  |  | *Penicillium commune* | GYA/PDA |
|  |  |  |  |  | *Pseudogymnoascus pannorum* | PDA |
|  |  |  |  |  | *Pyrenochaetopsis microspora* | PDA |
|  |  |  |  | 43 | *Acremonium fuci* | GYA |
|  |  |  |  |  | *Diaporthe* cf. *arecae* | GYA/PDA |
|  |  |  |  |  | *Diaporthe* cf. *sojae* | DRBC |
|  |  |  |  |  | *Epicoccum* cf. *duchesneae* | PDA |
|  |  |  |  |  | *Epicoccum duchesneae* | PDA |
|  |  |  |  |  | *Epicoccum sorghinum* | DRBC |
|  |  |  |  |  | *Kalmusia araucariae* | DRBC |
|  |  |  |  |  | *Neodidymelliopsis* cf. *longicolla* | DRBC |
|  |  |  |  |  | *Neopestalotiopsis* sp. | DRBC |
|  |  |  |  |  | *Paraphoma radicina* | DRBC |
|  |  |  |  |  | *Penicillium charlesii* | DRBC |
|  |  |  |  |  | *Penicillium commune* | GYA |
|  |  |  |  |  | *Pestalotiopsis* sp. | DRBC |
|  |  |  |  |  | *Phaeophleospora eucalypticola* | DRBC |
|  |  |  |  |  | *Phaeosphaeria oryzae* | PDA |
|  |  |  |  |  | Pleosporales sp. 1 | PDA |
|  |  |  |  |  | *Pseudogymnoascus pannorum* | DRBC |
|  |  |  |  |  | *Talaromyces rugulosus* | DRBC |
|  |  |  |  | 44 | *Aspergillus oryzae* | GYA |
|  |  |  |  |  | *Aspergillus tritici* | DRBC |
|  |  |  |  |  | *Botryosphaeria dothidea* | GYA |
|  |  |  |  |  | *Cladosporium* cf. *halotolerans* | DRBC |
|  |  |  |  |  | *Epicoccum* cf. *hordei* | PDA |
|  |  |  |  |  | *Epicoccum duchesneae* | GYA |
|  |  |  |  |  | *Neopestalotiopsis* sp. | PDA |
|  |  |  |  |  | *Penicillium commune* | DRBC/PDA |
|  |  |  |  |  | *Penicillium crustosum* | DRBC/GYA/PDA |
|  |  |  |  |  | *Penicillium exsudans* | PDA |
|  |  |  |  |  | *Penicillium oxalicum* | DRBC |
|  |  |  |  |  | *Pestalotiopsis* cf. *anacardiacearum* | PDA |
|  |  |  |  |  | *Pestalotiopsis* cf. *australasiae* | GYA/PDA |
|  |  |  |  |  | *Pestalotiopsis* sp. | DRBC/PDA |
|  |  |  |  |  | *Pestalotiopsis thailandica* | GYA |
|  |  |  |  |  | Pleosporales sp. 1 | GYA |
|  |  |  |  |  | Pleosporales sp. 3 | PDA |
|  |  |  |  |  | *Pyrenochaetopsis microspora* | GYA |
|  |  |  |  |  | *Sarocladium strictum* | DRBC/GYA |
| Site 15 | Mud | Songseok-ri, Maseo-myeon, Seocheon-gun, Chungcheongnam-do | 36.0816, 126.6250 | 45 | *Nothophoma quercina* | PDA |
|  |  |  |  | 46 | Pleosporaceae sp. | DRBC |
|  |  |  |  | 47 | Pleosporales sp. 2 | DRBC |

**Supplementary Table S2. Strains and their GenBank accession numbers used for phylogenetic analyses in this study.**

| **Species** | **Representative strain** | **ITS** | ***act*** | ***BenA*** |
| --- | --- | --- | --- | --- |
|  |  |  |  |  |
| *Acremonium* *fuci* | CMG27 | MK986700 | - | - |
|  | **SFC2022_NP060** | **OP070793** | - | - |
| *Acremonium* cf. *fuci* | **SFC2022_NP059** | **OP070792** | - | - |
| *Alternaria alternata* | CBS 127672 | MH864614 | - | - |
|  | **SFC2022_NP008** | **OP070736** | - | - |
| *Alternaria chlamydospora* | CBS 491.72 | NR_136039 | - | - |
|  | **SFC2022_NP054** | **OP070787** | - | - |
| *Alternaria* *rosae* | CBS 121341 | NR_136017 | - | - |
| *Alternaria* cf. *rosae* | **SFC2022_NP027** | **OP070755** | - | - |
| *Apiospora marii* | CBS 497.90 | NR_166043 | - | - |
|  | **SFC2022_NP020** | **OP070748** | - | - |
| *Apiospora rasikravindrae* | NFCCI 2144 | NR_119932 | - | - |
|  | **SFC20220715_M05** | **OP070781** | - | - |
| *Aspergillus ochraceus* | NRRL 398 | NR_077150 | - | EF661322 |
|  | **SFC2022_NP098** | **OP070834** | - | **OP022418** |
| *Aspergillus oryzae* | NRRL 447 | NR_135395 | - | EF661483 |
|  | **SFC2022_NP081** | **OP070817** | - | **OP022410** |
| *Aspergillus tritici* | CBS 266.81 | NR_135414 | - | MN969368 |
|  | **SFC2022_NP089** | **OP070825** | - | **OP022415** |
| *Aureobasidium melanogenum* | CBS 105.22 | NR_159598 | - | - |
|  | **SFC2022_NP040** | **OP070770** | - | - |
| *Aureobasidium namibiae* | CBS 147.97 | NR_147362 | - | - |
|  | **SFC2022_NP039** | **OP070769** | - | - |
| *Aureobasidium pullulans* | CBS 584.75 | NR_144909 | - | - |
|  | **SFC2022_NP046** | **OP070776** | - | - |
| *Botryosphaeria dothidea* | CMW 8000 | NR_111146 | - | - |
|  | **SFC2022_NP084** | **OP070820** | - | - |
| *Botrytis cinerea* | HNSMJ-4 | MW820601 | - | - |
|  | **SFC2022_NP013** | **OP070741** | - | - |
| *Cercospora beticola* | CBS 116456 | - | AY840458 | - |
| *Chaetomium globosum* | CBS 160.62 | NR_144851 | - | - |
|  | **SFC2022_NP028** | **OP070756** | - | - |
| *Cladosporium allicinum* | CBS 121624 | NR_152266 | EF679502 | - |
|  | **SFC2022_NP021** | **OP070749** | **OP022373** | - |
| *Cladosporium anthropophilum* | Clad 387 | - | MZ695001 | - |
|  | **SFC2022_NP015** | **OP070743** | **OP022371** | - |
| *Cladosporium* *cladosporioides* | CBS 112388 | NR_119839 | HM148490 | - |
| *Cladosporium* cf. *cladosporioides* | **SFC2022_NP022** | **OP070750** | **OP022374** | - |
| *Cladosporium funiculosum* | CBS 122129 | NR_119845 | HM148583 | - |
|  | **SFC20220715_M07** | **OP070802** | **OP022380** | - |
| *Cladosporium halotolerans* | CBS 119416 | NR_119605 | EF101397 | - |
|  | **SFC2022_NP036** | **OP070766** | **OP022375** | - |
| *Cladosporium* cf. *halotolerans* | **SFC2022_NP094** | **OP070830** | **OP022381** | - |
| *Cladosporium perangustum* | CBS 125996 | NR_119851 | HM148610 | - |
|  | **SFC2022_NP065** | **OP070798** | **OP022379** | - |
| *Cladosporium pseudocladosporioides* | CBS 125993 | NR_119852 | HM148647 | - |
|  | **SFC2022_NP037** | **OP070767** | **OP022376** | - |
| *Cladosporium ramotenellum* | CBS 121628 | NR_119658 | EF679538 | - |
|  | **SFC2022_NP063** | **OP070796** | **OP022378** | - |
| *Cladosporium rectoides* | CBS 125994 | NR_111539 | HM148683 | - |
|  | **SFC2022_NP016** | **OP070744** | **OP022372** | - |
| *Cladosporium tenuissimum* | CBS 125995 | NR_119855 | HM148687 | - |
|  | **SFC2022_NP009** | **OP070737** | **OP022370** | - |
| *Cladosporium xanthochromaticum* | UTHSC: DI13-211 | NR_148191 | LN834599 | - |
|  | **SFC2022_NP007** | **OP070735** | **OP022369** | - |
| *Cladosporium xylophilum* | CBS 125997 | NR_111541 | HM148721 | - |
|  | **SFC2022_NP051** | **OP070783** | **OP022377** | - |
| *Coniothyrium palmarum* | CBS 400.71 | MH860184 | - | KT389792 |
| *Cytospora ceratosperma* | CBS 116.21 | AY347335 | - | - |
|  | **SFC20220715_M02** | **OP070757** | - | - |
| *Diaporthe* *arecae* | CBS 161.64 | MH858400 | - | KC344000 |
| *Diaporthe* cf. *arecae* | **SFC2022_NP017** | **OP070745** | - | **OP022383** |
| *Diaporthe* *hungariae* | CBS 143353 | MG281126 | - | MG281299 |
| *Diaporthe* cf. *hungariae* | **SFC2022_NP026** | **OP070754** | - | **OP022387** |
| *Diaporthe* *pseudooculi* | HHUF 30617 | NR_161019 | - | LC373519 |
| *Diaporthe* cf. *pseudooculi* | **SFC2022_NP076** | **OP070812** | - | **OP022399** |
| *Diaporthe* *sojae* | CQHYD3-2 | MT877050 | - | MT874968 |
| *Diaporthe* cf. *sojae* | **SFC2022_NP099** | **OP070835** | - | **OP022405** |
| *Didymella macrophylla* | CGMCC 3.18357 | NR_158258 | - | KY742312 |
| *Didymella* cf. *macrophylla* | **SFC2022_NP004** | **OP070732** | - | **OP022382** |
| Didymosphaeriaceae sp. 1 | **SFC2022_NP096** | **OP070832** | - |  |
| Didymosphaeriaceae sp. 2 | **SFC2022_NP055** | **OP070788** | - | **OP022392** |
| *Epicoccum dendrobii* | CGMCC 3.18359 | NR_158261 | - | KY742335 |
|  | **SFC2022_NP049** | **OP070780** | - | **OP022389** |
| *Epicoccum duchesneae* | CGMCC 3.18345 | NR_158262 | - | KY742337 |
|  | **SFC2022_NP077** | **OP070813** | - | **OP022400** |
| *Epicoccum* cf. *duchesneae* | **SFC2022_NP078** | **OP070814** | - | **OP022401** |
| *Epicoccum hordei* | CGMCC 3.18360 | NR_158263 | - | KY742339 |
| *Epicoccum* cf. *hordei* | **SFC2022_NP073** | **OP070807** | - | **OP022397** |
| *Epicoccum nigrum* | CBS 173.73 | MH860655 | - | FJ427107 |
| *Epicoccum sorghinum* | UTHSC: DI16-301 | LT592948 | - | LT593017 |
|  | **SFC2022_NP079** | **OP070815** | - | **OP022419** |
| *Epicoccum* cf. *sorghinum* | **SFC2022_NP023** | **OP070751** | - | **OP022385** |
| *Epicoccum* sp. | **SFC2022_NP052** | **OP070785** | - | **OP022391** |
| *Epicoccum tritici* | MFLUCC-16-0276 | KX926426 | - | KY197979 |
|  | **SFC2022_NP019** | **OP070747** | - | **OP022384** |
| *Eutypella* cf. *persica* | IRAN 2540C | NR_171807 | - | - |
|  | **SFC2022_NP032** | **OP070761** | - | - |
| *Fusarium equiseti* | NRRL 26419 | NR_121457 | - | - |
|  | **SFC2022_NP053** | **OP070786** | - | - |
| *Fusarium fujikuroi* | CBS 221.76 | NR_111889 | - | - |
|  | **SFC2022_NP044** | **OP070774** | - | - |
| *Hamigera avellanea* | CBS 295.48 | - | - | LC076692 |
| Hypocreales sp. | **SFC2022_NP033** | **OP070762** | - | - |
| *Juxtiphoma eupyrena* | CBS 832.84 | MH859842 | - | MN983994 |
| *Juxtiphoma* cf. *eupyrena* | **SFC2022_NP067** | **OP070800** | - | **OP022394** |
| *Kalmusia araucariae* | CPC 37475 | NR_170054 | - | - |
|  | **SFC20220715_M08** | **OP070808** | - | - |
| *Morinia acaciae* | CBS 137994 | NR_161082 | - | MH554673 |
| *Morinia* cf. *acaciae* | **SFC2022_NP075** | **OP070811** | - | - |
| *Neocamarosporium betae* | WW18CQ02 | MZ734407 | - | - |
|  | **SFC2022_NP005** | **OP070733** | - | - |
| *Neocamarosporium leipoldtiae* | CBS 146774 | NR_171762 | - | - |
| *Neocamarosporium solicola* | IBRC M 30257 | KX817217 | - | - |
|  | **SFC2022_NP041** | **OP070771** | - | - |
| *Neocamarosporium* sp. | **SFC2022_NP042** | **OP070772** | - | - |
| *Neodevriesia metrosideri* | CBS 145084 | NR_161141 | - | - |
| *Neodevriesia* cf. *metrosideri* | **SFC2022_NP048** | **OP070779** | - | - |
| *Neodidymelliopsis longicolla* | CBS 382.96 | KT389532 | - | KT389830 |
|  | **SFC2022_NP024** | **OP070752** | - | **OP022386** |
| *Neodidymelliopsis* cf. *longicolla* | **SFC2022_NP088** | **OP070824** | - | **OP022402** |
| *Neopestalotiopsis rosae* | CBS 101057 | NR_145243 | - | KM199429 |
| *Neopestalotiopsis* sp. | **SFC2022_NP069** | **OP022396** | - | - |
| *Neosetophoma poaceicola* | MFLUCC 16-0886 | NR_165861 | - | - |
|  | **SFC20220715_M06** | **OP070784** | - | - |
| *Neosetophoma* cf. *poaceicola* | **SFC2022_NP018** | **OP070746** | - | - |
| *Neosetophoma rosigena* | MFLU 17-0626 | NR_157525 | - | - |
|  | **SFC20220715_M03** | **OP070764** | - | - |
| *Nigrospora cf. oryzae* | CBS 480.73 | NR_153476 | - | - |
|  | **SFC2022_NP034** | **OP070763** | - | - |
| *Nothophoma quercina* | CBS 832.84 | - | - | MN983992 |
|  | **SFC2022_NP038** | **OP070768** | - | **OP022388** |
| *Paracamarosporium hawaiiense* | CBS 120025 | NR_154287 | - | - |
| *Paraconiothyrium brasiliense* | CBS 100299 | NR_163552 | - | - |
|  | **SFC2022_NP043** | **OP070773** | - | - |
| *Paradendryphiella arenariae* | CBS 181.58 | NR_145170 | - | - |
|  | **SFC2022_NP066** | **OP070799** | - | - |
| *Paraphoma radicina* | CBS 111.79 | NR_156556 | - | - |
|  | **SFC2022_NP087** | **OP070823** | - | - |
| *Parasarocladium gamsii* | CBS 726.71 | NR_159615 | - | - |
|  | **SFC20220715_M04** | **OP070778** | - | - |
| *Parathyridaria tyrrhenica* | MUT 5371 | NR_169907 | - | - |
| *Parathyridaria* cf. *tyrrhenica* | **SFC2022_NP080** | **OP070816** | - | - |
| *Parengyodontium album* | CBS 504.83 | LC092880 | - | - |
|  | **SFC2022_NP030** | **OP070759** | - | - |
| *Penicillium charlesii* | CBS 304.48 | AF033400 | - | JX091508 |
|  | **SFC2022_NP082** | **OP070818** | - | **OP022411** |
| *Penicillium commune* | CBS 311.48 | AY213672 | - | MN969377 |
|  | **SFC2022_NP092** | **OP070828** | - | **OP022417** |
| *Penicillium crustosum* | CBS 115503 | AF033472 | - | MN969379 |
|  | **SFC2022_NP091** | **OP070827** | - | **OP022416** |
| *Penicillium echinulatum* | CBS 317.48 | AF033473 | - | AY674341 |
|  | **SFC2022_NP058** | **OP070791** | - | **OP022409** |
| *Penicillium expansum* | CBS 325.48 | AY373912 | - | AY674400 |
|  | **SFC2022_NP056** | **OP070789** | - | **OP022408** |
| *Penicillium exsudans* | CGMCC 3.18412 | KX885062 | - | KX885042 |
|  | **SFC2022_NP085** | **OP070821** | - | **OP022413** |
| *Penicillium javanicum* | CBS 341.48 | GU981613 | - | GU981657 |
|  | **SFC2022_NP010** | **OP070738** | - | **OP022406** |
| *Penicillium oxalicum* | CBS 219.30 | AF033438 | - | KF296462 |
|  | **SFC2022_NP086** | **OP070822** | - | **OP022414** |
| *Penicillium roqueforti* | CBS 221.30 | HQ442347 | - | MN969396 |
|  | **SFC2022_NP012** | **OP070740** | - | **OP022407** |
| *Pestalotiopsis anacardiacearum* | IFRDCC 2397 | NR_120255 | - | KC247155 |
| *Pestalotiopsis* cf. *anacardiacearum* | **SFC2022_NP090** | **OP070826** | - | **OP022403** |
| *Pestalotiopsis australasiae* | CBS 114126 | NR_147546 | - | KM199409 |
| *Pestalotiopsis* cf. *australasiae* | **SFC2022_NP050** | **OP070782** | - | **OP022390** |
| *Pestalotiopsis* sp. | **SFC2022_NP074** | **OP070809** | - | **OP022398** |
| *Pestalotiopsis thailandica* | MFLUCC 17-1616 | NR_164471 | - | MK764352 |
|  | **SFC2022_NP097** | **OP070833** | - | **OP022404** |
| *Phaeophleospora eucalypticola* | CPC 26523 | NR_145123 | - | - |
|  | **SFC20220715_M09** | **OP070810** | - | - |
| *Phaeosphaeria culmorum* | CBS 570.86 | MH861992 | - | - |
|  | **SFC2022_NP006** | **OP070734** | - | - |
| *Phaeosphaeria oryzae* | CBS 110110 | MH862850 | - | - |
|  | **SFC2022_NP095** | **OP070831** | - | - |
| *Phaeosphaeria spartinicola* | CBS 176.91 | MH862249 | - | - |
|  | **SFC2022_NP029** | **OP070758** | - | - |
| *Plectosphaerella cucumerina* | CBS 131739 | NR_171712 | - | - |
|  | **SFC2022_NP002** | **OP070758** | - | - |
| *Pleosporaceae* sp. 1 | **SFC2022_NP031** | **OP070760** | - | - |
| *Pleosporaceae* sp. 2 | **SFC2022_NP047** | **OP070777** | - | - |
| Pleosporales sp. 1 | **SFC2022_NP014** | **OP070742** | - | - |
| Pleosporales sp. 2 | **SFC2022_NP035** | **OP070765** | - | - |
| Pleosporales sp. 3 | **SFC2022_NP070** | **OP070804** | - | - |
| Pleosporales sp. 4 | **SFC2022_NP001** | **OP070728** | - | - |
| *Pseudogymnoascus pannorum* | CBS 106.13 | MH866140 | - | - |
|  | **SFC2022_NP072** | **OP070806** | - | - |
| *Pyrenochaetopsis microspora* | CBS 102876 | NR_160059 | - | - |
|  | **SFC2022_NP093** | **OP070829** | - | - |
| *Pyrenochaetopsis paucisetosa* | NNIBRFG27317 | MW041623 | - | - |
|  | **SFC2022_NP025** | **OP070753** | - | - |
| *Remotididymella capsici* | CBS 679.77 | MN973478 | - | MT005578 |
|  | **SFC2022_NP061** | **OP070794** | - | **OP022393** |
| *Remotididymella* sp. | **SFC2022_NP068** | **OP070801** | - | **OP022395** |
| *Sarocladium strictum* | CBS 346.70 | NR_111145 | - | - |
|  | **SFC2022_NP071** | **OP070805** | - | - |
| *Sedecimiella taiwanensis* | MUT<ITA>:5053 | KR014368 | - | - |
|  | **SFC2022_NP003** | **OP070731** | - | - |
| *Septoriella hubertusii* | CBS 338.86 | NR_155786 | - | - |
| *Septoriella* cf. *hubertusii* | **SFC2022_NP057** | **OP070790** | - | - |
| *Sphaeropsis sapinea* | CBS 393.84 | NR_152452 | - | - |
|  | **SFC2022_NP045** | **OP070775** | - | - |
| *Stemphylium lycopersici* | CBS 122639 | NR_155002 | - | - |
|  | **SFC2022_NP062** | **OP070795** | - | - |
| *Stemphylium vesicarium* | CBS 109844 | MH862840 | - | - |
|  | **SFC2022_NP011** | **OP070739** | - | - |
| *Talaromyces rugulosus* | CBS 371.48 | NR_103676 | - | KF984575 |
|  | **SFC2022_NP083** | **OP070819** | - | **OP022412** |
| *Trichoderma fomiticola* | CBS 121136 | NR_134391 | - | - |
|  | **SFC20220715_M01** | **OP070730** | - | - |
| *Trichoderma harzianum* | CBS 226.95 | AY605713 | - | - |
|  | **SFC2022_NP064** | **OP070797** | - | - |
| *Valsa ambiens* | CFCC 89894 | - | - | KU710989 |

**Supplementary Table S3. Clear zone data for all tested fungal strains.** The degradation abilities are categorized into four levels.

| **Species** | **Representative**  **strains** | **Clear Zone Length (mm)** | **Degradation ability*** |
| --- | --- | --- | --- |
|  |  |  |  |
| *Acremonium* cf. *fuci* | SFC2022_NP059 | 0.00 | (0) |
|  |  | 0.00 | (0) |
|  |  | 0.00 | (0) |
| *Acremonium fuci* | SFC2022_NP060 | 0.00 | (0) |
|  |  | 0.00 | (0) |
| *Alternaria alternata* | SFC2022_NP008 | 5.78 | (++) |
|  |  | 12.88 | (+++) |
|  |  | 5.03 | (++) |
|  |  | 9.28 | (++) |
|  |  | 3.32 | (+) |
|  |  | 3.76 | (+) |
|  |  | 4.34 | (+) |
|  |  | 4.73 | (+) |
| *Alternaria* cf. *rosae* | SFC2022_NP027 | 5.82 | (++) |
| *Alternaria chlamydospora* | SFC2022_NP054 | 1.70 | (+) |
| *Apiospora marii* | SFC2022_NP020 | 0.51 | (+) |
| *Apiospora rasikravindrae* | SFC20220715_M05 | 0.85 | (+) |
| *Aspergillus ochraceus* | SFC2022_NP098 | 2.19 | (+) |
| *Aspergillus oryzae* | SFC2022_NP081 | 0.85 | (+) |
| *Aspergillus tritici* | SFC2022_NP089 | 2.03 | (+) |
| *Aureobasidium melanogenum* | SFC2022_NP040 | 8.28 | (++) |
| *Aureobasidium namibiae* | SFC2022_NP039 | 6.24 | (++) |
| *Aureobasidium pullulans* | SFC2022_NP046 | 0.56 | (+) |
| *Botryosphaeria dothidea* | SFC2022_NP084 | 0.37 | (+) |
| *Botrytis cinerea* | SFC2022_NP013 | 1.37 | (+) |
| *Chaetomium globosum* | SFC2022_NP028 | 0.00 | (0) |
| *Cladosporium allicinum* | SFC2022_NP021 | 13.92 | (+++) |
| *Cladosporium anthropophilum* | SFC2022_NP015 | 9.74 | (++) |
| *Cladosporium* cf. *halotolerans* | SFC2022_NP094 | 8.60 | (++) |
| *Cladosporium* cf. *cladosporioides* | SFC2022_NP022 | 8.22 | (++) |
| *Cladosporium funiculosum* | SFC20220715_M07 | 1.66 | (+) |
| *Cladosporium halotolerans* | SFC2022_NP036 | 2.46 | (+) |
| *Cladosporium perangustum* | SFC2022_NP065 | 3.80 | (+) |
|  |  | 9.72 | (++) |
| *Cladosporium pseudocladosporioides* | SFC2022_NP037 | 2.90 | (+) |
|  |  | 12.57 | (+++) |
| *Cladosporium ramotenellum* | SFC2022_NP063 | 5.70 | (++) |
|  |  | 5.32 | (++) |
|  |  | 7.26 | (++) |
|  |  | 1.60 | (+) |
|  |  | 2.27 | (+) |
|  |  | 3.70 | (+) |
| *Cladosporium rectoides* | SFC2022_NP016 | 8.02 | (++) |
|  |  | 12.65 | (+++) |
| *Cladosporium tenuissimum* | SFC2022_NP009 | 10.83 | (+++) |
|  |  | 10.52 | (+++) |
|  |  | 9.28 | (++) |
| *Cladosporium xanthochromaticum* | SFC2022_NP007 | 11.37 | (+++) |
| *Cladosporium xylophilum* | SFC2022_NP051 | 0.65 | (+) |
|  |  | 0.49 | (+) |
| *Cytospora ceratosperma* | SFC20220715_M02 | 6.17 | (++) |
| *Diaporthe* cf. *arecae* | SFC2022_NP017 | 1.05 | (+) |
|  |  | 1.61 | (+) |
| *Diaporthe* cf. *hungariae* | SFC2022_NP026 | 3.15 | (+) |
|  |  | 1.80 | (+) |
| *Diaporthe* cf. *pseudooculi* | SFC2022_NP076 | 2.18 | (+) |
| *Diaporthe* cf. *sojae* | SFC2022_NP099 | 1.09 | (+) |
| *Didymella* cf. *macrophylla* | SFC2022_NP004 | 1.28 | (+) |
| Didymosphaeriaceae sp. 1 | SFC2022_NP096 | 6.10 | (++) |
| Didymosphaeriaceae sp. 2 | SFC2022_NP055 | 1.13 | (+) |
| *Epicoccum* cf. *duchesneae* | SFC2022_NP078 | 3.58 | (+) |
| *Epicoccum* cf. *hordei* | SFC2022_NP073 | 0.00 | (0) |
| *Epicoccum* cf. *sorghinum* | SFC2022_NP023 | 2.65 | (+) |
| *Epicoccum dendrobii* | SFC2022_NP049 | 6.26 | (++) |
| *Epicoccum duchesneae* | SFC2022_NP077 | 0.86 | (+) |
|  |  | 0.00 | (0) |
| *Epicoccum sorghinum* | SFC2022_NP079 | 1.50 | (+) |
| *Epicoccum* sp. | SFC2022_NP052 | 0.35 | (+) |
| *Epicoccum tritici* | SFC2022_NP019 | 2.34 | (+) |
| *Eutypella* cf. *persica* | SFC2022_NP032 | 0.00 | (0) |
| *Fusarium equiseti* | SFC2022_NP053 | 0.48 | (+) |
|  |  | 0.78 | (+) |
| *Fusarium fujikuroi* | SFC2022_NP044 | 0.73 | (+) |
| Hypocreales sp. | SFC2022_NP033 | 2.43 | (+) |
| *Juxtiphoma* cf. *eupyrena* | SFC2022_NP067 | 0.00 | (0) |
| *Kalmusia araucariae* | SFC20220715_M08 | 0.00 | (0) |
| *Morinia* cf. *acaciae* | SFC2022_NP075 | 1.55 | (+) |
| *Neocamarosporium betae* | SFC2022_NP005 | 0.00 | (0) |
| *Neocamarosporium solicola* | SFC2022_NP041 | 0.55 | (+) |
| *Neocamarosporium* sp. | SFC2022_NP042 | 1.37 | (+) |
|  |  | 0.35 | (+) |
| *Neodevriesia* cf. *metrosideri* | SFC2022_NP048 | 6.08 | (++) |
| *Neodidymelliopsis* cf. *longicola* | SFC2022_NP088 | 0.00 | (0) |
|  |  | 6.42 | (++) |
|  |  | 1.42 | (+) |
| *Neodidymelliopsis longicolla* | SFC2022_NP024 | 0.77 | (+) |
| *Neopestalotiopsis* sp. | SFC2022_NP069 | 0.00 | (0) |
| *Neosetophoma* cf. *poaceicola* | SFC2022_NP018 | 2.31 | (+) |
| *Neosetophoma poaceicola* | SFC20220715_M06 | 0.94 | (+) |
| *Neosetophoma rosigena* | SFC20220715_M03 | 0.58 | (+) |
| *Nigrospora* cf. *oryzae* | SFC2022_NP034 | 0.00 | (0) |
| *Nothophoma quercina* | SFC2022_NP038 | 7.67 | (++) |
| *Paraconiothyrium brasiliense* | SFC2022_NP043 | 0.00 | (0) |
| *Paradendryphiella arenariae* | SFC2022_NP066 | 0.00 | (0) |
|  |  | 0.00 | (0) |
|  |  | 0.52 | (+) |
| *Paraphoma radicina* | SFC2022_NP087 | 0.77 | (+) |
| *Parasarocladium* cf. *gamsii* | SFC20220715_M04 | 0.49 | (+) |
|  |  | 6.26 | (++) |
| *Parathyridaria* cf. *tyrrhenica* | SFC2022_NP080 | 0.00 | (0) |
|  |  | 1.46 | (+) |
| *Parengyodontium album* | SFC2022_NP030 | 0.00 | (0) |
| *Penicillium charlesii* | SFC2022_NP082 | 0.00 | (0) |
| *Penicillium commune* | SFC2022_NP092 | 0.00 | (0) |
|  |  | 0.00 | (0) |
| *Penicillium crustosum* | SFC2022_NP091 | 0.00 | (0) |
| *Penicillium echinulatum* | SFC2022_NP058 | 0.37 | (+) |
| *Penicillium expansum* | SFC2022_NP056 | 0.00 | (0) |
| *Penicillium exsudans* | SFC2022_NP085 | 0.00 | (0) |
| *Penicillium javanicum* | SFC2022_NP010 | 0.00 | (0) |
| *Penicillium oxalicum* | SFC2022_NP086 | 0.00 | (0) |
| *Penicillium roqueforti* | SFC2022_NP012 | 0.07 | (+) |
| *Pestalotiopsis* cf. *anacardiacearum* | SFC2022_NP090 | 0.88 | (+) |
| *Pestalotiopsis* cf. *australasiae* | SFC2022_NP050 | 0.34 | (+) |
| *Pestalotiopsis* sp. | SFC2022_NP074 | 0.90 | (+) |
| *Pestalotiopsis thailandica* | SFC2022_NP097 | 1.35 | (+) |
| *Phaeophleospora eucalypticola* | SFC20220715_M09 | 13.96 | (+++) |
| *Phaeosphaeria culmorum* | SFC2022_NP006 | 0.00 | (0) |
| *Phaeosphaeria spartinicola* | SFC2022_NP029 | 5.27 | (++) |
| *Phaeosphaeria oryzae* | SFC2022_NP095 | 6.42 | (++) |
| *Plectosphaerella cucumerina* | SFC2022_NP002 | 0.39 | (+) |
| Pleosporaceae sp. | SFC2022_NP031 | 0.42 | (+) |
| Pleosporaceae sp. 2 | SFC2022_NP047 | 2.07 | (+) |
| Pleosporales sp. 1 | SFC2022_NP014 | 0.00 | (0) |
|  |  | 0.00 | (0) |
| Pleosporales sp. 2 | SFC2022_NP035 | 0.52 | (+) |
|  |  | 2.14 | (+) |
| Pleosporales sp. 3 | SFC2022_NP070 | 0.93 | (+) |
| Pleosporales sp. 4 | SFC2022_NP001 | 2.29 | (+) |
|  |  | 4.44 | (+) |
| *Pseudogymnoascus pannorum* | SFC2022_NP072 | 0.89 | (+) |
| *Pyrenochaetopsis microspora* | SFC2022_NP093 | 1.24 | (+) |
| *Pyrenochaetopsis paucisetosa*. | SFC2022_NP025 | 0.69 | (+) |
| *Remotididymella* cf. *capsici* | SFC2022_NP061 | 0.00 | (0) |
|  |  | 1.02 | (+) |
| *Remotiodidymella* sp. | SFC2022_NP068 | 2.78 | (+) |
| *Sarocladium strictum* | SFC2022_NP071 | 7.63 | (++) |
| *Sedecimiella taiwanensis* | SFC2022_NP003 | 0.19 | (+) |
| *Septoriella* cf. *hubertusii* | SFC2022_NP057 | 0.65 | (+) |
| *Sphaeropsis sapinea* | SFC2022_NP045 | 6.94 | (++) |
| *Stemphylium lycopersici* | SFC2022_NP062 | 1.00 | (+) |
| *Stemphylium vesicarium* | SFC2022_NP011 | 1.61 | (+) |
|  |  | 1.71 | (+) |
| *Talaromyces rugulosus* | SFC2022_NP083 | 3.31 | (+) |
| *Trichoderma fomiticola* | SFC20220715_M01 | 0.30 | (+) |
| *Trichoderma harzianum* | SFC2022_NP064 | 0.41 | (+) |

* 0 mm: (0), 0 < (+) ≤ 5 mm, 5 < (++) ≤ 10 mm, 10 < (+++) ≤ 15 mm
